# Scale‐dependent environmental effects on phenotypic distributions in *Heliconius* butterflies

**DOI:** 10.1002/ece3.9286

**Published:** 2022-09-13

**Authors:** Ananda R. Pereira Martins, Lucas P. Martins, Wing‐Zheng Ho, William Owen McMillan, Jonathan S. Ready, Rowan Barrett

**Affiliations:** ^1^ Redpath Museum McGill University Montreal Quebec Canada; ^2^ Smithsonian Tropical Research Institute Panama City Panama; ^3^ School of Biological Sciences University of Canterbury Christchurch New Zealand; ^4^ Instituto de Ciências Biológicas Universidade Federal do Pará Belém Brazil

**Keywords:** adaptation, aposematism, color pattern, Müllerian mimicry, multiscale distribution, phenotypic diversity

## Abstract

Identifying the relative importance of different mechanisms responsible for the emergence and maintenance of phenotypic diversity can be challenging, as multiple selective pressures and stochastic events are involved in these processes. Therefore, testing how environmental conditions shape the distribution of phenotypes can offer important insights on local adaptation, divergence, and speciation. The red‐yellow Müllerian mimicry ring of *Heliconius* butterflies exhibits a wide diversity of color patterns across the Neotropics and is involved in multiple hybrid zones, making it a powerful system to investigate environmental drivers of phenotypic distributions. Using the distantly related *Heliconius erato* and *Heliconius melpomene* co‐mimics and a multiscale distribution approach, we investigated whether distinct phenotypes of these species are associated with different environmental conditions. We show that *Heliconius* red‐yellow phenotypic distribution is strongly driven by environmental gradients (especially thermal and precipitation variables), but that phenotype and environment associations vary with spatial scale. While co‐mimics are usually predicted to occur in similar environments at large spatial scales, patterns at local scales are not always consistent (i.e., different variables are best predictors of phenotypic occurrence in different locations) or congruent (i.e., co‐mimics show distinct associations with environment). We suggest that large‐scale analyses are important for identifying how environmental factors shape broad mimetic phenotypic distributions, but that local studies are essential to understand the context‐dependent biotic, abiotic, and historical mechanisms driving finer‐scale phenotypic transitions.

## Graphical abstract

Using a multiscale approach and different *Heliconius* butterfly phenotypes, we investigated how the environment shapes the distribution of mimetic wing color patterns and assessed the overlap of co‐mimics' distribution.

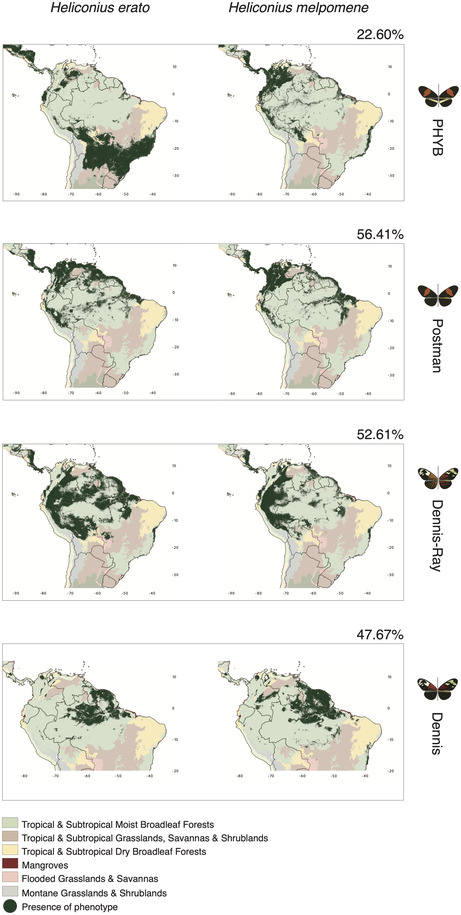

## 1 INTRODUCTION

Investigating how environmental conditions influence the distribution of species and their populations can provide important insights into the generation and maintenance of phenotypic diversity (Edelaar, 2018; Gould & Johnston, 1972; Schluter, 2001). Indeed, numerous studies have documented local phenotypic adaptation in response to habitat heterogeneity (e.g., Barrett et al., 2019; Boncoraglio & Saino, 2007; Hegna et al., 2013). However, it has long been recognized that a combination of selective forces and stochastic events may influence the spatial distribution of phenotypes (Amézquita et al., 2009; McLean & Stuart‐Fox, 2014). Accordingly, disentangling the factors promoting phenotypic diversity remains a complex task, as these mechanisms can directly or indirectly influence each other (Gray & McKinnon, 2007; Nosil, 2012).

*Heliconius* butterflies represent an excellent system for exploring phenotypic diversity and identifying the environmental factors influencing phenotypic distributions. Distantly related *Heliconius* species (along with other butterfly and moth genera) have converged on similar aposematic wing colors, forming a handful of local mimicry rings (Birskis‐Barros et al., 2021; Mallet, 1999; Papageorgis, 1975; Turner, 1971). Interestingly, the presence of various aposematic rings in a single area contradicts predictions from the Müllerian mimicry theory, which posits that local phenotypic convergence resulting in shared warning patterns should reduce the cost of predator learning (Joron & Mallet, 1998; Mallet, 1993; Sherratt, 2008). This system also presents a remarkable variation of wing color patterns within a single mimicry ring, such that populations of a species characterized by specific colors (such as red, yellow, and black) can have very different patterns, resulting in patchworks of distinct phenotypes across the range of a species (Brown Jr. et al., 1974; Papageorgis, 1975; Turner, 1971).

While previous research has shown that wing color in *Heliconius* populations are under strong natural and sexual selection (Dell'Aglio et al., 2016, 2018; Jiggins et al., 2001, 2004; Mallet & Barton, 1989; Merrill et al., 2012, 2014), the environmental factors driving the spatial distribution of color phenotypes among and within mimicry rings remain unclear (Gompert et al., 2011; Joron, 2005; Joron & Mallet, 1998; Mallet, 1993). More specifically, although some studies have tested the influence of environmental gradients on *Heliconius* phenotypes (Arias et al., 2008; Blum, 2008; Jiggins et al., 1996; Rosser et al., 2014; Thurman et al., 2019), most large‐scale distribution maps are based solely on occurrence points, without environmental variables being used as predictors (Brown Jr. et al., 1974; Brown Jr., 1982; Rosser et al., 2012; Turner, 1971). Additionally, previous distribution modeling of *Heliconius* species has not accounted for intraspecific phenotypic variation (Rueda‐M et al., 2021). This is relevant because distinct phenotypes usually have varying fitness advantage depending on the location (Arias et al., 2008; Blum, 2008), leading to differences in their geographical distributions. Finally, no study has investigated the associations among environmental variables and *Heliconius* phenotypes across distinct spatial scales, despite a growing recognition that the relative influence of environmental predictors may be scale‐dependent (Bunnell & Huggard, 1999; Sandel & Smith, 2009; Wiens & Bachelet, 2010).

**FIGURE 1:**
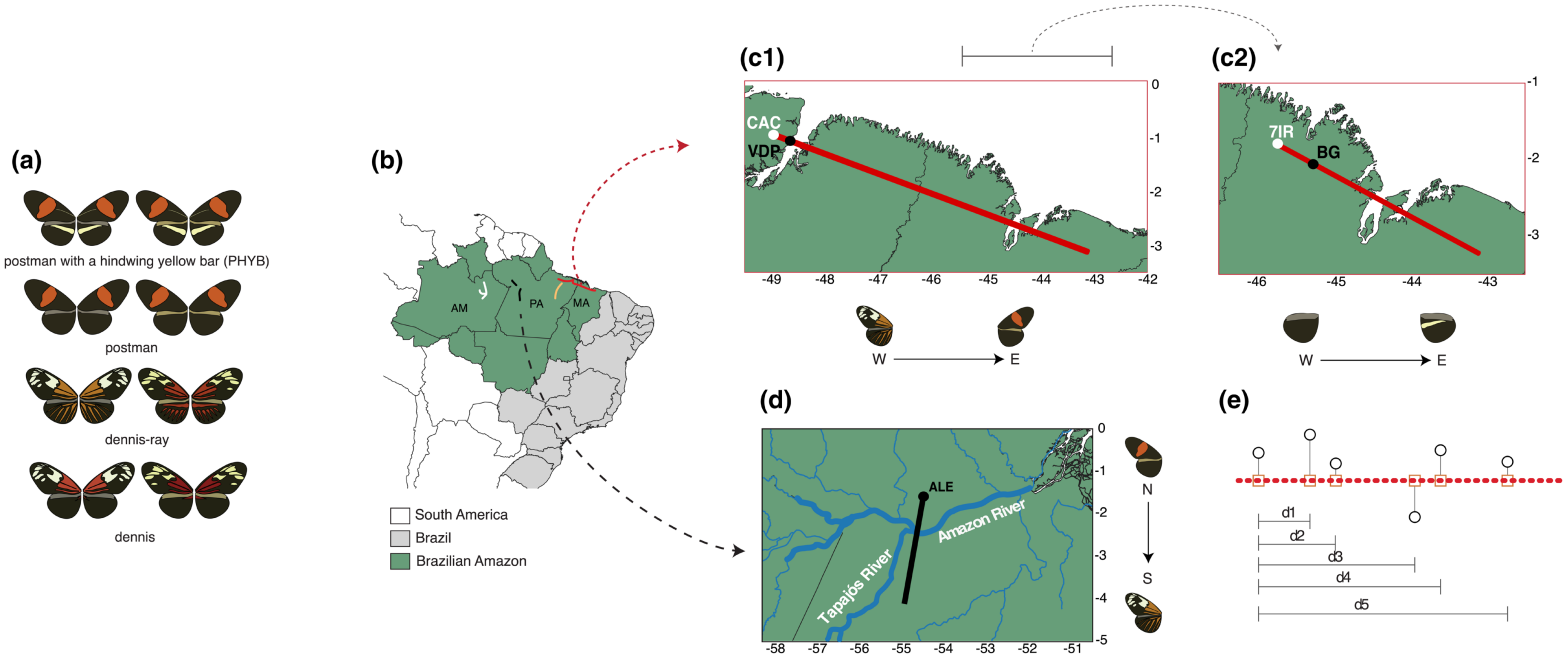
Phenotypes of the red‐yellow Müllerian mimicry ring and transects across the Brazilian Amazon. (a) For each phenotype, left represents *Heliconius erato* morphs and right represents *H. melpomene* morphs. (b) Transects represented by red, yellow, black, and white lines located in three states of Brazil: Maranhão (MA), Pará (PA), and Amazonas (AM). (c–d) Two transects were used in our cline analysis, as these crossed hybrid zones (dashed arrows in ‘b’). Black dots and IDs within transects represent the westernmost or northmost common collection site of *H. erato* and *H. melpomene*. White dots and IDs within transects represent the westernmost collection site of *H. melpomene*. (c1) Transition 1 moving from West to East and following the change from dennis‐ray (*H. e. amazona*, *H. e. estrella/H. m. thelxiope*, *H. m. madeira*, *H. m. intersectus*) to postman phenotype (*H. e. hydara/H. m. melpomene*). (c2) Transition 2 following the change from absence of a hindwing yellow bar (postman: *H. e. hydara/H. m. melpomene*) to the presence of the bar (PHYB: *H. e. phyllis/H. m. burchelli*, *H. m. nanna*). (d) Transition 3 moving from North to South and crossing the Amazon River, following the change from postman (*H. e. hydara/H. m. melpomene*) to dennis‐ray (*H. e. amazona*, *H. e. lativitta/H. m. madeira*). (e) Schematics showing how distances along the transects are calculated. Distances ‘d’ are calculated relative to the westernmost or northmost collection sites.

In this study, we estimated the spatial distribution of *Heliconius* phenotypes within a single mimicry ring, using two distantly related and non‐hybridizing co‐mimics, *Heliconius erato* and *Heliconius melpomene*. These two species are part of the red‐yellow mimicry ring [red and yellow group according to Turner, 1971], which shows a remarkable phenotypic variation in red, yellow and orange wing elements across the Neotropics (Figure 1a). We used a multiscale approach including a large‐scale analysis covering the entire distribution of the co‐mimics, as well as a local‐scale analysis focusing on hybrid zones within the Brazilian Amazon Forest. The Amazon region contains some of the most biodiverse terrestrial ecosystems (Da Silva et al., 2005; Myers et al., 2000), which may be partially attributed to high variation in precipitation, seasonality, and temperature (Hijmans et al., 2005). Our local‐scale sampling covers regions explored by the naturalist Henry Walter Bates over 150 years ago. Notably, Bates postulated that the geographical turnover in *H. melpomene* phenotypes he observed during his explorations was the result of variation in humidity, vegetation, and soil types (Bates, 1862).

By analyzing the effect of different environmental variables on the distribution of the distinct phenotypes belonging to the red‐yellow mimicry ring (Figure 1a) at multiple scales, we aim to answer: (i) Is there a general relationship between *Heliconius* wing color phenotypes and environmental variables across the Neotropics? (ii) How congruent are the predictive variables between co‐mimics? (iii) If phenotype–environment associations exist, are they consistent across distinct spatial scales? In addition, we aim to find support for two hypotheses based on different mechanisms of selection: (1) *The Signaling hypothesis*, which predicts that phenotypes with larger melanized wing areas are associated with open‐canopy forests and higher solar irradiation, since darker aposematic patterns are more conspicuous against lighter environment backgrounds; accordingly, we expect that paler phenotypes should be found in denser forests, as these patterns better advertise aposematism in shaded environments (Arenas et al., 2014; Osorio & Vorobyev, 2005; Rojas et al., 2018; Stevens, 2007); and (2) *The Thermoregulation hypothesis*, which predicts that darker phenotypes are associated with cooler environments with lower solar irradiation, as darker wings provide greater efficiency for thermoregulation; accordingly, we expect that less melanized phenotypes should be found in warmer habitats with higher solar irradiation, as these would be more efficient at avoiding overheating (Clusella‐Trullas et al., 2007; Clusella‐Trullas & Nielsen, 2020; Hegna et al., 2013; Van Dyck & Matthysen, 1998).

## 2 MATERIAL AND METHODS

### 2.1 Large‐scale analysis (distribution models)

We used Species Distribution Modeling (SDM; Guisan & Thuiller, 2005) to predict the distribution of *H. erato* and *H. melpomene* phenotypes within the red‐yellow Müllerian mimicry ring (Figure 1a). SDM is an approach that combines known coordinate occurrences with information about the environmental tolerances of a species to map suitable habitats (Austin, 2002; Elith & Leathwick, 2009). We sampled butterflies using entomological nets from December 2016 to June 2017 and from July to October 2018 at ~25 km intervals across four transects in the Brazilian Amazon, totaling 77 sites distributed across ~1905 km (Figure 1b; Table S1). Transects were selected to capture a wide array of ecosystems (Figure S1) and variation in environmental and physical features across the Amazon, as well as to include regions containing hybrid zones within the two studied species. We also included samples from the collection of the Laboratory of Ecology and Systematics of Pollinators and Predators (LESPP—Federal University of Maranhão) and data used in Rosser et al. (2012), available at https://heliconius‐maps.github.io/. For each species, subspecies belonging to the same phenotype were considered as a single group to produce a predicted distribution map (Table S2). In total, we used 3403 occurrence points after filtering for duplicated records, uncertainty or incomplete taxon identification, and grids without environmental data (Figure S2).

Our environmental dataset comprised 31 variables selected based on their potential direct and indirect effects on *Heliconius* ecology and evolution (Table S3). Selected variables were prepared using QGIS (QGIS Association, 2020) with a 1 km resolution. To avoid collinearity and model overfitting, we performed Pearson's correlation and selected variables using a cut‐off of *r* < .7 (Dormann et al., 2013). Our final dataset comprised 12 environmental variables, including climate, soil, vegetation, wind, and solar parameters (Table S3).

We performed distribution modeling using the Maximum Entropy Method (MaxEnt; Phillips et al., 2006) with the SDMtune package (Vignali et al., 2020) in R (R Core Team, 2019). Maxent is a machine learning method that outperforms other distributional modeling techniques based on different metrics that assess the agreement between records and predictions (Elith et al., 2006; Phillips et al., 2006; Wisz et al., 2008). Maxent does not require true absence data, which is rarely available (Elith et al., 2006; Phillips et al., 2006), and its results can be interpreted as habitat suitability for taxa based on a set of environmental predictors (Phillips et al., 2006). For background data, we randomly created 10,000 points covering the entire distribution of *H. erato* and *H. melpomene* (Barbet‐Massin et al., 2012). We randomly selected 75% of presence and background data for training, and 25% for testing models' predictability. Additionally, we used a 10‐fold cross‐validation test as a validation strategy (Braunisch & Suchant, 2010) and performed the fine‐tuning strategy to select Maxent's best parameters (Vignali et al., 2020; Table S4). This method allowed us to achieve the best predictive power, while avoiding model overfitting (Hallgren et al., 2019; Radosavljevic & Anderson, 2014).

We used the Area Under the Curve of the Receiver Operating Characteristic (AUC) and the True Skill Statistic (TSS) as evaluation metrics, considering an AUC > 0.7 and a TSS > 0.5 as a non‐random distribution model with respect to the predictors (Allouche et al., 2006; Elith et al., 2006). To evaluate the importance of each environmental variable as a model's predictor, we used the permutation importance metric. This was calculated by permuting one variable at a time (using 10 repetitions) and computing the decrease in the model's AUC metric. Excluded variables that mostly decreased AUC values were considered more important to the model (Vignali et al., 2020).

We converted predicted distributions into binary maps using the maximum sensitivity plus specificity as threshold (Liu et al., 2013, 2016). The raster R package (Hijmans & van Etten, 2020) was used to calculate the intersection area in the distribution of co‐mimics and Logistic Maxent response curves were used to evaluate the probability of a phenotype being present in a locality (Merow et al., 2013).

Finally, we overlapped the predicted distribution of each phenotype with a map of the world's biomes (Dinerstein et al., 2017) to evaluate whether changes in phenotypes are associated with biome boundaries. As biomes represent global‐scale ecosystems characterized by unique sets of environmental conditions (Hoekstra et al., 2004; Olson et al., 2001), we expected to find a pattern of changes in phenotypes across biome boundaries.

### 2.2 Local‐scale analysis

To test the effects of local‐scale environmental gradients on phenotypic distributions, we performed cline analyses along the two transects in the Brazilian Amazon in which intermediate phenotypes were sampled (Figures 1b–d and S1). Importantly, we conducted color variation tests prior to cline analysis to evaluate whether the four previously identified phenotypes (Figure 1a) formed distinct clusters based on color patterns. We took high‐resolution photos of *H. erato* and *H. melpomene* subspecies representing the four phenotypes (Figure 1a; Table S5) and performed a Principal Component Analysis (PCA) with the patternize R package (Van Belleghem, Papa, et al., 2018). To assess differences in black patterning among phenotypes, we estimated the wing surface area containing black scales in the different clusters using one‐way ANOVA and Tukey's tests (Figure S3).

For our local‐scale analysis, we focused on hybrid zones involving pure phenotypes that presented distinct black patterning (higher melanin x lower melanin). Using this approach, we were able to test opposing hypotheses about mechanisms of selection from visual signaling versus thermoregulation. Importantly, we could not perform this analysis in our large‐scale approach because we did not have a scenario in which there were clear transitions of morphs showing a significant difference in black patterning (Figure S3).

We performed cline analyses using a Bayesian approach implemented in the bahz R package (Thurman, 2019). Geographic coordinates were transformed using linear regression to obtain one‐dimensional transects, adapting a method described in Thurman et al. (2019). The “new” coordinates were set as the closest point within the linear transect to the true location (Figure 1e), and distances were calculated using two approaches: (1) relative to the westernmost or northmost sampling site for each species, which were not necessarily the same as we were not able to collect both species in all the sites and (2) relative to the westernmost or northmost common site for both co‐mimics.

Two transitions of phenotypes were evaluated in cline analyses. The first transition represented the transition from dennis‐ray to postman (or vice‐versa), crossing hybrid zones in which samples presented variable intermediate phenotypes of red patterns and are treated as heterozygotes (in contrast to the parental forms, which are treated as homozygotes). Two transects contained transitions involving these two phenotypes, one located in the Eastern Brazilian Amazon (Figure 1c1), and one located in the Central Brazilian Amazon, close to the Amazon and Tapajós rivers' confluence (Figure 1d). The second phenotypic transition was analyzed using a subset of the first transect (Figure 1c2), following the transition from the absence of a hindwing yellow bar (postman) to the presence of the bar (PHYB), crossing a hybrid zone in which hybrids present a faint bar, visible as a shadow on the ventral hindwing (Mallet, 1986) and/or sparse yellow scales on the dorsal hindwing.

We used cline results to overlay hybrid zones on a map of terrestrial ecoregions (Dinerstein et al., 2017) to test whether changes in phenotypes are associated with ecoregion boundaries. Ecoregions are defined as regional‐scale ecosystems nested within biomes (Hoekstra et al., 2004; Olson et al., 2001); as such, ecoregions represent ecological boundaries at finer scales when compared with biomes.

Finally, to directly evaluate whether changes in phenotypic frequencies at local scales are associated with environmental variables, we performed spearman correlations using seven environmental parameters, including climate, solar and vegetation variables (Table S3). Only variables with pairwise correlations lower than 0.7 (to avoid collinearity; Dormann et al., 2013) and that were found to be important in the best‐fitting distribution models in our large‐scale analysis were selected.

## 3 RESULTS

### 3.1 Abiotic variables predict large‐scale phenotypic distributions

**FIGURE 2:**
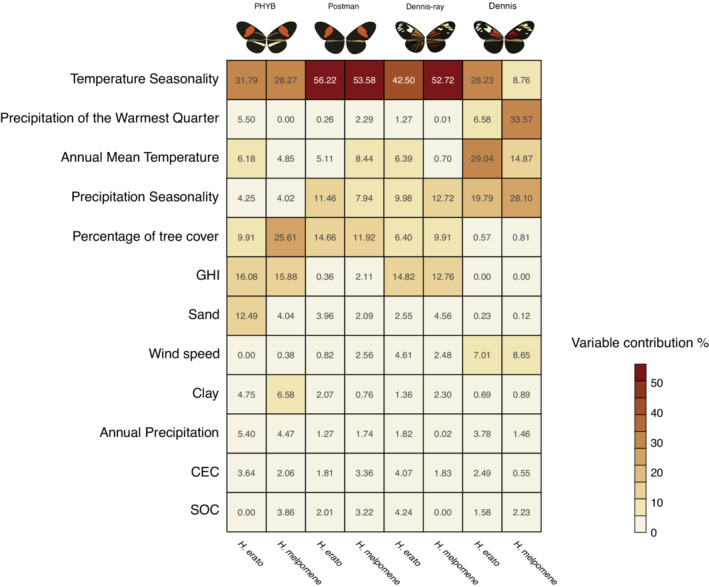
Environmental variables and their contribution (in percent) towards predicting phenotypic distributions. Each column represents a species, as indicated at the bottom of the figure. Shared phenotypes are indicated by pairs of columns at the top of the figure.

We found that environmental variables play an important role in driving the large‐scale distribution of phenotypes within the *Heliconius* red‐yellow mimicry ring, as indicated by the high values of our models' predictive performance (AUC > 0.8 and TSS > 0.5, Table S6). The variable with the most explanatory power for *Heliconius* phenotypic distributions was temperature seasonality, representing the highest contribution for PHYB (*H. erato*: 31.79% and *H. melpomene*: 28.27%), postman (*H. erato*: 56.22% and *H. melpomene*: 53.58%), and dennis‐ray (*H. erato*: 42.50% and *H. melpomene*: 52.72%) models (Figure 2). The exception was the dennis phenotype, in which annual mean temperature was the most important predictor for *H. erato* (29.04%), and precipitation of the warmest quarter (33.57%) for *H. melpomene*. However, temperature seasonality is still among the most important predictors for dennis models, being the second highest contribution for *H. erato* (28.23%) and the fourth highest contribution for *H. melpomene* (8.76%; Figure 2).

The rank order of contributions by environmental predictors showed that thermal and precipitation seasonality are particularly important for predicting phenotypic distributions of *H. erato* and *H. melpomene* co‐mimics, although precipitation seasonality had lower relative importance for PHYB. Percentage of tree cover and solar irradiation (GHI) were also important for predicting distributions, although less important for the dennis phenotype (Figure 2). In general, soil parameters and wind speed had low contributions in most models.

**FIGURE 3:**
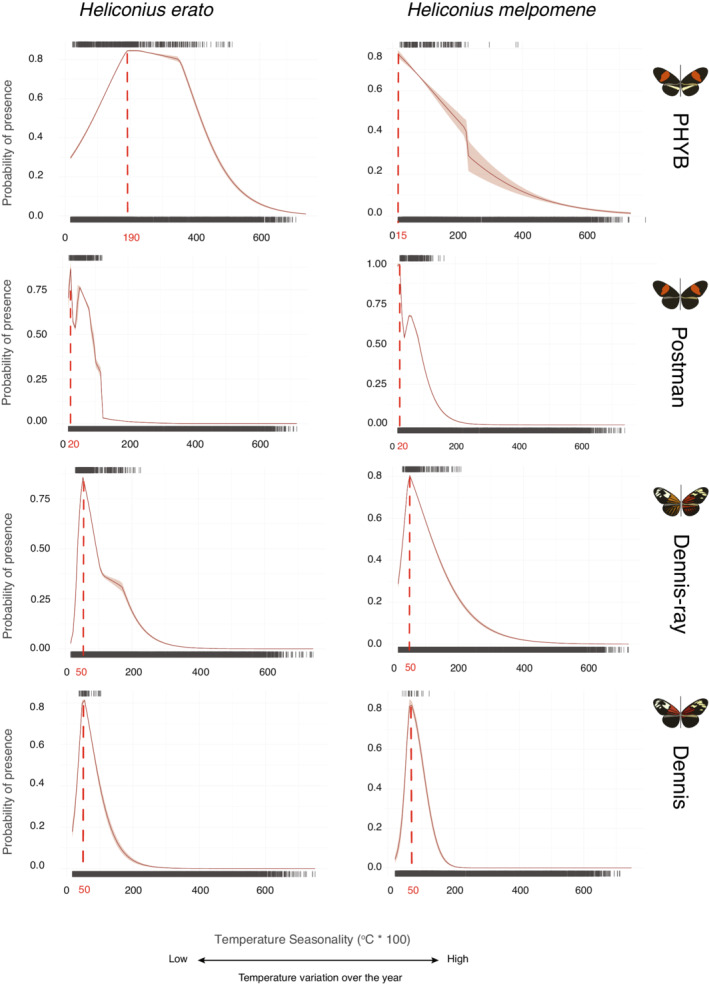
Logistic Maxent response curves showing how the probability of presence of *Heliconius erato* and *H. melpomene* phenotypes varies along the temperature seasonality gradient. Here, temperature seasonality is measured as the standard deviation of monthly temperature averages throughout 1979–2013 (Karger & Zimmermann, 2019; O'Donnell & Ignizio, 2012). Environmental optima values associated with higher probability of presence are shown as dashed red lines. Each row indicates a different phenotype, with the left column representing *H. erato* and the right column representing *H. melpomene*. Red shading around response curves shows 95% confidence intervals.

**FIGURE 4:**
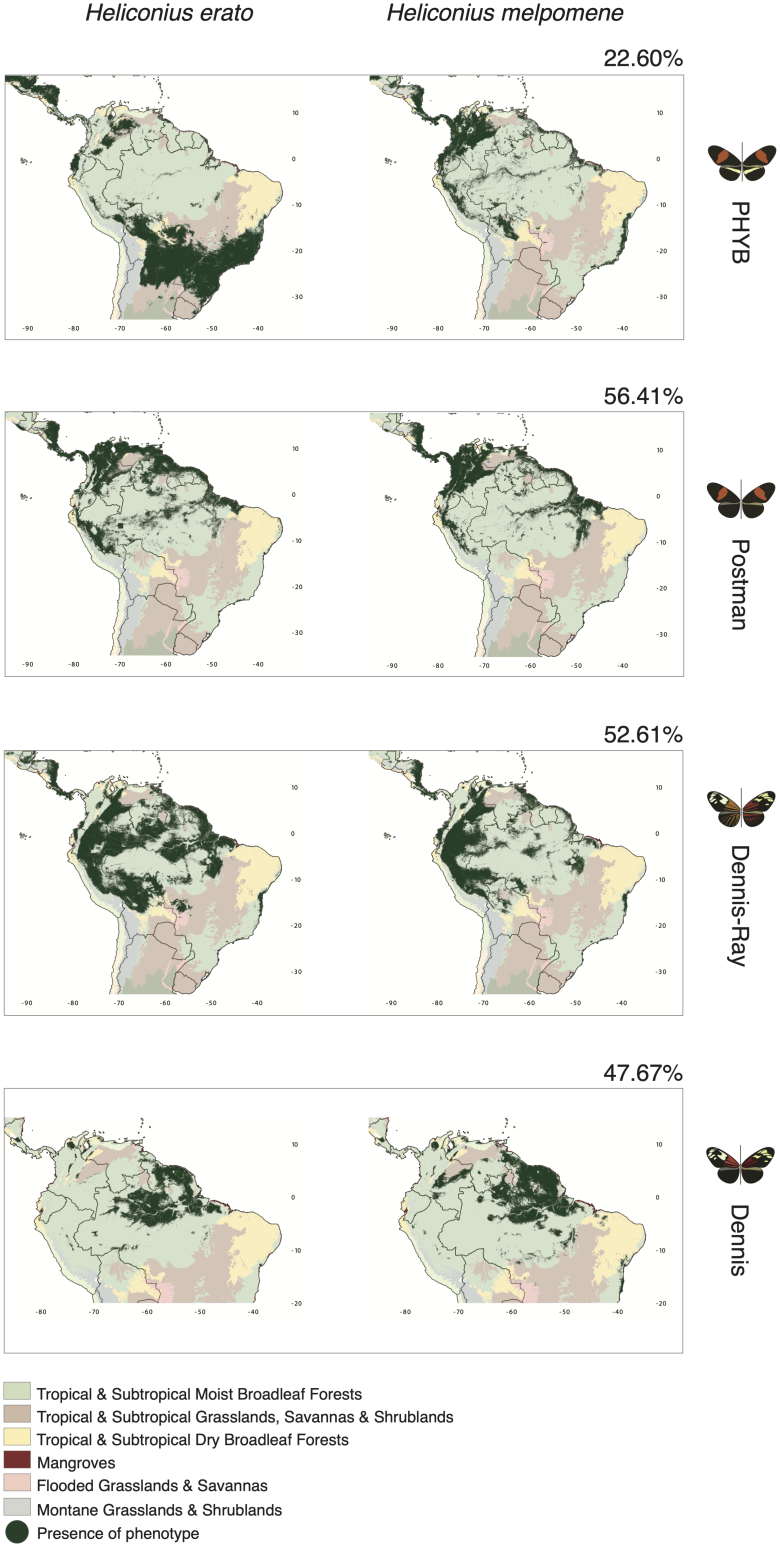
*Heliconius erato* and *H. melpomene* phenotypic predicted distribution maps relative to major biomes. The overlap in area between distributions of the co‐mimics is indicated as a percentage at the top right for each phenotype. For each phenotype, the map on the left represents *H. erato* and the map on the right represents *H. melpomene*. The biomes’ map is available at http://ecoregions2017.appspot.com.

Response curves (i.e., the probability of a phenotypic presence as a function of an environmental gradient) indicate that environmental predictors are more similar between co‐mimics than among phenotypes within the same species (Figures 3 and S4). For instance, two thermal variables (temperature seasonality and annual mean temperature) showed distinct responses among phenotypes but generally similar optima values and response curves between co‐mimics in three out of the four co‐mimetic phenotypes. The exception was the PHYB phenotype, in which the phenotypic optimum between co‐mimics were highly different. Our maps also showed considerable overlap (>47%) in the predicted distributions of co‐mimics, except for the PHYB phenotype (22.6%; Figure 4). Interestingly, even though several environmental variables contributed to explaining the large‐scale distribution of *Heliconius* phenotypes, we did not find abrupt changes in phenotype composition across biome boundaries. In fact, our models suggest that all phenotypes occur in more than one biome (Figure 4). Notably, the postman phenotype was associated with warmer environments and lower temperature seasonality when compared with the dennis and dennis‐ray phenotypes (Figures 3 and S4).

Although co‐mimics usually had similar response curve shapes, they do not necessarily have the same optimum environmental value [i.e., the predictor value(s) associated with the highest probability of presence, according to ter Braak & Looman, 1986]. Percentage of tree cover and solar irradiation (GHI) had different optima values between co‐mimics for all four phenotypes (Figure S4). Additionally, even when there was congruence of optima values and curve shapes (Figure 3), there were often differences in the variation around the optimum value between co‐mimics.

### 3.2 Effects of abiotic gradients on local phenotypic transitions

Consistent with the broad scale patterns, we found that local phenotypic transitions are not associated with ecoregion boundaries. Nonetheless, in contrast to our large‐scale results, our local‐scale analyses revealed incongruencies in co‐mimic relationships with environmental gradients and inconsistent environmental effects in different regions of the Amazon.

**FIGURE 5:**
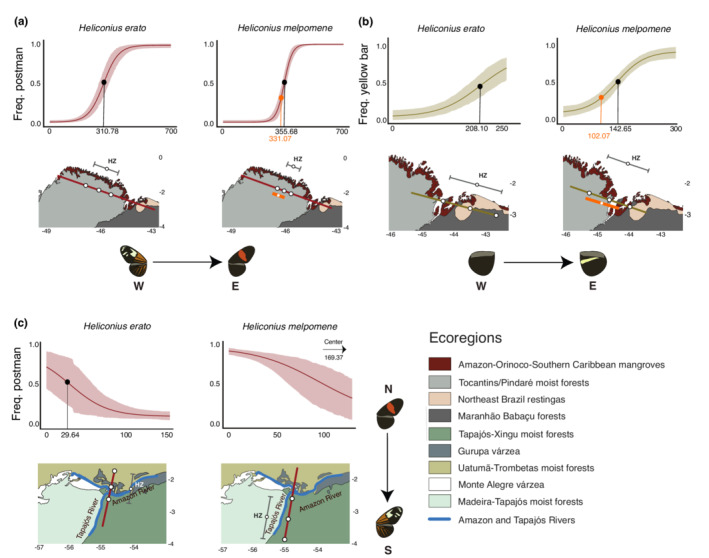
Local‐scale cline analyses. Black dots within cline figures indicate the center of hybrid zones. Orange dots within cline figures indicate the center of *H. melpomene* hybrid zones, considering the westernmost shared collection site with *H. erato*. Hybrid zone limits and centers are indicated by three white dots within the maps and the letters HZ. Orange lines within maps represent *H. melpomene* hybrid zones, considering the westernmost shared collection site with *H. erato*. (a) dennis‐ray to postman transitions. (b) postman to PHYB transitions. (c) postman to dennis‐ray transitions. Cline figures: 95% confidence intervals.

**FIGURE 6:**
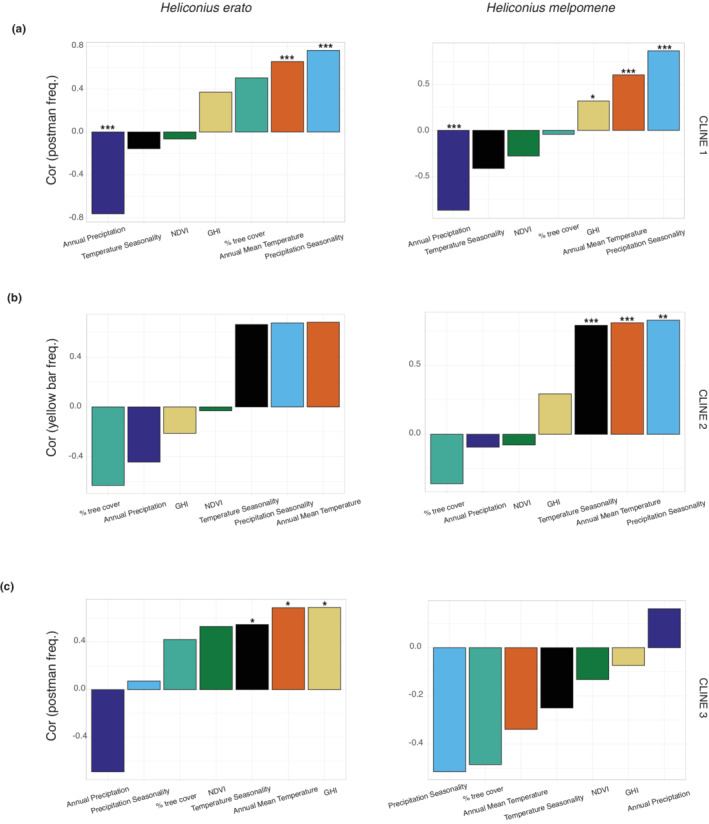
Correlations between postman or hindwing yellow bar frequencies and environmental variables along hybrid zones (Table S3). (a) Correlations between the postman frequency and environmental variables across the first transition (dennis‐ray to postman phenotype). (b) Correlations between the hindwing yellow bar frequency and environmental variables across the second transition (postman to PHYB phenotype). (c) Correlations between the postman frequency and environmental variables across the third transition (postman to dennis‐ray phenotype). *p*‐values: 0 ‘***’ .001 ‘**’ .01 ‘*’ .05.

In transition 1 (Figure 1c1), we observed a similar shape and location for hybrid zones of both *H. erato* and *H. melpomene*. This transition followed the change from dennis‐ray (a lower melanin morph—Figure S3) to postman (a higher melanin morph—Figure S3). The predicted center of the *H. erato* hybrid zone is 310.78 km from the westernmost collection site and has a predicted width of 173.68 km (Figure 5a; Table S7). *H. melpomene* has a steeper hybrid zone with a center at 355.68 km from the westernmost collection site and has a width of 93.74 km (Figure 5a; Table S7). Taking into consideration the common westernmost site for both co‐mimics, the center of the *H. melpomene* hybrid zone is at 331.07 km and has a width of 92.66 km (Figures 5a and S5; Table S7). Both hybrid zones are entirely located within a single ecoregion (Figure 5a), indicating that changes in ecoregions are not associated with this phenotypic transition. However, changes in specific environmental variables show congruent associations with phenotypic transitions between co‐mimics. We found that the frequency change in the postman phenotype across the hybrid zone for both species and environmental variables indicate that compared with the lower melanin dennis‐ray phenotype, the higher melanin postman phenotype is associated with higher mean temperatures, higher precipitation seasonality, and higher solar irradiation, as well as lower annual mean precipitation, lower temperature seasonality, and lower vegetation density (Figure 6a; Table S8).

Transition 2 (Figure 1c2) occurs in the same region as transition 1 and follows the change from postman phenotype (a higher melanin morph—Figure S3) to the PHYB phenotype (a lower melanin morph—Figure S3). Within this phenotypic transition, there is incongruence between the co‐mimic hybrid zones. The predicted center of the *H. erato* hybrid zone is at 208.10 km from the westernmost site and has a predicted width of 194.08 km, crossing three different ecoregions (Figure 5b; Table S7), while the predicted center of the *H. melpomene* hybrid zone is at 142.65 km from the westernmost site, crossing two ecoregions (Figure 5b). *H. melpomene* also has a steeper hybrid zone compared with *H. erato*, with a predicted width of 149.13 km (Figure 5b, Table S7). Considering the common westernmost site for both co‐mimics, the center of the *H. melpomene* hybrid zone is at 102.07 km and has a width of 144.32 km (Figures 5b and S5; Table S7). As in transition 1, changes in specific environmental variables showed congruent associations with phenotypic transitions between co‐mimics, as demonstrated by the similar pattern of correlations between the frequency change in the hindwing yellow bar and environmental variables in *H. erato* and *H. melpomene* (Figure 6b; Table S8). However, within these phenotypic transitions we found that, compared with the lower melanin PHYB phenotype, the higher melanin postman phenotype is associated with lower annual mean temperature, temperature seasonality, and precipitation seasonality (Figure 6b; Table S8).

Finally, transition 3 (Figure 1d), which followed the change from postman (higher melanin morph—Figure S3) to dennis‐ray (lower melanin morph—Figure S3) phenotypes, showed distinct co‐mimic associations and hybrid zone locations. The predicted center of the *H. erato* hybrid zone is 29.64 km from the northmost collection site. This hybrid zone has a predicted width of 95.21 km, crossing three different ecoregions and the Amazon River (Figure 5c; Table S7). In comparison, the predicted center of the *H. melpomene* hybrid zone is 169.37 km from the northmost collection site and is less steep than the *H. erato* hybrid zone, with a predicted width of 132.53 km, being entirely located within a single ecoregion (Figure 5c). Importantly, within this transition, *H. erato* and *H. melpomene* shared the northmost collection site. In addition to differences in co‐mimic hybrid zone locations, the associations with abiotic variables were not congruent between co‐mimics, as shown by the contrasting directions in the correlations between the postman frequency and the environmental variables in *H. erato* and *H. melpomene* (Figure 6c; Table S8). Furthermore, the strengths of correlations are distinct between co‐mimics. For example, precipitation seasonality has a weak positive correlation with the postman frequency in *H. erato* but presents the strongest negative correlation in *H. melpomene*. The statistically significant correlations were all positive and found in *H. erato*. In particular, a strong positive correlation for GHI in *H. erato* indicates its importance as a local distribution predictor, while it shows no significant correlation in *H. melpomene*.

## 4 DISCUSSION

Our multiscale approach revealed that phenotypic distributions were driven by environmental variables (predominantly climatic variables associated to heat and pluviometry seasonality), with congruent patterns between most *H. erato* and *H. melpomene* co‐mimics across the Neotropics. However, we found more nuanced patterns at finer spatial scales, with phenotype–environment associations being congruent between co‐mimics in some hybrid zones and incongruent in others, and with inconsistent phenotype–environment associations in distinct regions of the Brazilian Amazon. Thus, although we find evidence that environmental forces are shaping the distribution of phenotypic variation over large spatial scales (Amiot et al., 2021; Connor et al., 2019), local forces, not necessarily linked to climate variables, can override and blur the effects of environmental selection.

### 4.1 Phenotypic distribution across the neotropics

At a broad spatial scale, phenotypic distributions were mostly explained by temperature seasonality and to a lesser extent by precipitation seasonality. It is likely that seasonality directly affects *Heliconius* distributions due to the association between wing color and physiological tolerance, as has been shown for other tropical butterfly species (Dongmo et al., 2021; Fischer & Kirste, 2018; Silva et al., 2020). Indeed, differences in black color patterns confer distinct thermoregulation effects in ectotherms (Clusella‐Trullas & Nielsen, 2020), and melanin has a protective role against desiccation (King & Sinclair, 2015; Parkash et al., 2009; Parkash, Rajpurohit, & Ramniwas, 2009; Rajpurohit et al., 2008). In particular, Wasserthal (1983) showed a one‐way flow of hemolymph from the wing veins towards the membrane and suggested that melanin enhanced this flow as evaporation increased. However, there is currently no evidence linking specific color patterns within *H. erato* and *H. melpomene* to higher or lower tolerance of heat and/or dry conditions. Seasonality may also have an indirect effect on *Heliconius* distributions due to effects on hostplant distributions and abundance, as well as on *Heliconius* hostplant preference range (Benson, 1978; Jorge et al., 2011; Merrill et al., 2013; Rodrigues & Moreira, 2002, 2004). Even if the abiotic conditions are suitable for a given *Heliconius* phenotype, populations may not persist locally because of a lack of suitable hostplants (Araújo & Luoto, 2007; Lemoine, 2015).

Our large‐scale findings also indicate that the postman phenotype is predicted to occur in warmer regions with more constant temperatures compared with the dennis‐ray and dennis phenotypes. Interestingly, we found no congruent patterns for the postman with a yellow hindwing bar phenotype (PHYB), with distinct distributions and associations with environmental gradients between co‐mimics. More specifically, higher probabilities of occurrence in *H. erato* PHYB are associated with a wider range of precipitation values when compared with *H. melpomene*. This corroborates with *H. erato* PHYB being present in both Grasslands and Savannas as well as in Moist Broadleaf Forests biomes, whereas the distribution of *H. melpomene* PHYB is more restricted to the latter. Importantly, differences in large‐scale phenotypic distributions could also be a consequence of distinct colonization and/or extinction histories (Brower, 1996; Joron & Mallet, 1998; Mallet et al., 1996; Quek et al., 2010; Turner & Mallet, 1996).

### 4.2 Local phenotypic distributions

Environmental variables were also important in predicting local distributional patterns in most of the transition zones we investigated. However, at the local level there were differences in the congruence of environmental effects between co‐mimics and in the consistency of phenotype–environment associations across distinct hybrid zones. In the two hybrid zones located in the Eastern Amazon, we found that co‐mimics share relatively similar shaped transitions (i.e., curves showing phenotypic frequency change along the transect) and that those transitions were associated with similar environmental variables. However, the specific environmental features differed among these hybrid zones. The dennis‐ray to postman transition shows greater congruence between co‐mimics than the postman to PHYB transition. In contrast, there was no congruence between co‐mimics in the third hybrid zone located in the center of the Amazon. This is true for both the phenotypic frequency curve shapes and the associations with different environmental variables.

A number of factors may be interacting and are likely important in shaping local‐scale distributions of species and populations. These include local selective pressures (Lenormand et al., 2009; McLean & Stuart‐Fox, 2014), geological history (Hall & Harvey, 2002), shared ancestry (Losos, 2008; Wiens & Graham, 2005), different diversification histories (Quek et al., 2010), and stochastic events causing genetic drift (Mallet, 1993, 2010; Sherratt, 2006). These factors are probably interacting across the area of the Amazon basin that we studied. At the population level, frequency‐dependent selection (FDS) by predators, a selection regime in which the fitness of a local phenotype increases with its frequency (reviewed in Ruxton et al., 2004; Sherratt, 2008), is thought to be among the most important forces influencing aposematic pattern distributions in *Heliconius* (Finkbeiner et al., 2014; Kapan, 2001; Mallet & Barton, 1989). The magnitude of FDS is influenced by the presence of different predators (Nokelainen et al., 2014; Willmott et al., 2017), their distinct visual systems (Dell'Aglio et al., 2018), and innate differences in their learning and memory abilities (Endler & Mappes, 2004; Mappes et al., 2005; Speed & Turner, 1999), as well as the prey density (Kapan, 2001) and the dispersal of the butterflies (Barton & Hewitt, 1985, 1989). In this light, one might predict that selection on wing patterns that are more effective visual signals in a given environment might explain local phenotypic transitions. However, of the three transitions zones that we assayed, only one is consistent with this hypothesis. The postman phenotype possesses higher percentage of wings covered with black scales (proxy for the amount of melanin) than the dennis‐ray phenotype and would be a better aposematic signal in warmer, brighter and open habitats (Arenas et al., 2014; Osorio & Vorobyev, 2005; Rojas et al., 2018; Stevens, 2007). The transition from dennis‐ray to postman phenotype, located in Eastern Amazon, is consistent with this expectation. Indeed, molecular variation around genomic regions responsible of red color patterns suggests that the dennis‐rayed phenotype arose recently in both *H. erato* and *H. melpomene* and spread across the Amazon basin replacing the more ancestral postman phenotype (Hines et al., 2011). The hypothesized spread of this phenotype could be due to its better signaling ability in moist Amazonian forests. This phenotype is part of a much larger mimicry ring composed of over a dozen different butterfly and moth species (Papageorgis, 1975; Turner, 1971). Thus, a combination of signal efficiency and an experienced predator community could be responsible for this association. The genetic evidence for widespread phenotypic replacement remains speculative and our phenotypic data is only correlational. Thus, we cannot exclude the action of other selective pressures or random processes (Cuthill et al., 2017; Postema et al., 2022; Schluter et al., 1991) for the contemporary phenotypic distributional patterns. Moreover, in a hybrid zone approximately 1100 km away and involving the same phenotypes, there was no compelling support for the signaling hypothesis. Here, the *H. erato* and *H. melpomene* transition zones were disjunct. The *H. erato* zone was centered on the Amazon River, which is a potent, albeit often semipermeable, dispersal barrier for many species (Godinho & da Silva, 2018; Hayes & Sewlal, 2004; Lynch Alfaro et al., 2015; Rosser et al., 2020; Smith et al., 2014). In contrast, the transition between *H. melpomene* phenotypes was located approximately 120 km south of the Amazon River and largely uncorrelated with environmental variables. These discordances probably reflect different dispersal histories (Quek et al., 2010; Van Belleghem, Baquero, et al., 2018).

Given the broadscale association of phenotypes with environmental variables, it also seems likely that differences in the ability for different wing pattern phenotypes to thermoregulate may help explain local distributional patterns. However, again the results of our local scale analysis were mixed, with only one transition zone being roughly consistent with differences in thermoregulation. Here, the PHYB phenotype (i.e., postman with a hindwing yellow bar) is mostly associated with warmer environments, where its paler wings (compared with the postman phenotype) might provide a more efficient way to avoid overheating (Clusella‐Trullas et al., 2007; Clusella‐Trullas & Nielsen, 2020; Hegna et al., 2013; Van Dyck & Matthysen, 1998). Although slightly geographically offset, the hybrid zones of co‐mimics are nevertheless congruent in their environmental predictors. Interestingly, this transition zone crosses a bay in the north of the Maranhão state, with *H. melpomene* hybrid zone dislocated to West in comparison to *H. erato's*. Hence, similar to our transect in the center of the Amazon, differences in the dispersal history may have affected the distributions of phenotypes resulting in distinct co‐mimic hybrid zone locations.

A consistent theme in prior hybrid zone work is the suggestion of a role of the environment in shaping distributional patterns (Arias et al., 2008; Benson, 1982; Jiggins et al., 1996; Muñoz et al., 2010). Accordingly, even if other factors show to have greater effects on hybrid zone locations (such as the balance of predation and dispersal), direct and/or indirect habitat effects cannot be completely dismissed (Mallet, 1993; Mallet & Barton, 1989). For example, Blum (2008), showed that color pattern genotype and phenotype frequencies corresponded to land cover differences in a French Guiana *H. erato* hybrid zone, supporting the hypothesis of visual signaling efficiency for mate choice. On the other hand, Mallet and Barton (1989) suggested that patterns of hybrid zones of *H. erato* and *H. melpomene* in Peru are primarily explained by predation; however, they did not discard subspecies adaptations to local ecological conditions. Thus, although the specific environmental mechanisms and the strength of their effects vary across localities, there is abundant evidence for the environment acting as an important driver of local patterns of phenotypic distribution.

*Heliconius* phenotypic variation associated with habitat characteristics can also be explored under the context of spatial–temporal dynamics, as hybrid zones can move in response to environmental gradients (Barton, 1979; Barton & Hewitt, 1985; Buggs, 2007; Mallet, 1986, 1993; Wielstra, 2019). Blum (2002), for example, suggested that deforestation could be driving the movement of a *H. erato* hybrid zone in Panama, which was characterized 17 years earlier (Mallet, 1986). This zone between a postman (*H. e. hydara*) and a PHYB (*H. e. demophoon*) subspecies continued to move over the next 15 years, but at a slower rate (Thurman et al., 2019). Thurman et al. did not find a strong association between movement and deforestation, suggesting that the genetically dominant postman is replacing the PHYB phenotype through a phenomenon referred to as “dominance‐drive” (see Mallet, 1986). However, the authors could not rule out the importance of environment and associated predator community as also being responsible for the changing distributions of phenotype through time. Of course, hybrid zones can also be stable. For example, Rosser et al. (2014) showed that *H. erato* and *H. melpomene* clines in PHYB and dennis/rayed in Peru were stable over a 25 year time period despite substantial deforestation across the area. Here, the phenotypic transition occurs in an area of high rain fall and, although the authors could not rule out local adaptation due to climatic conditions, they posited that the transition zone was stable because of low population density of both species in the region of very high precipitation. Therefore, the majority of emerging data, including this study, supports the role of the environment in shaping patterns of underlying genetic and associated phenotypic variation, even though other factors can interact and/or mask its effect.

One of the most compelling aspects of studying the distribution of phenotypes are the implications for local adaptation and ecological speciation (Nosil, 2012). Here, studies focusing on different stages of speciation and investigating varying scales of environmental adaptation are important to understand how biodiversity arises (Harrison & Larson, 2014; Jiggins & Mallet, 2000; Larson et al., 2014). McMillan et al. (1997), for example, suggested that adaptation to wet and dry forests along with strong mate preference and FDS by predators was important in speciation between *H. erato* and *H. himera*. More recent work on this pair of incipient species demonstrated genetic differences in flight height and foraging behaviors associated with adaptation to different habitat characteristics (Dell'Aglio et al., 2022). Similarly, Muñoz et al. (2010) showed combination of pre‐ and post‐mating isolation mechanisms are important in the early stages of speciation in *H. erato* subspecies, *H. e. chestertonii* and *H. e. venus*. The two subspecies occasionally hybridize in a narrow transition zone associated with a strong (wet/dry) environmental gradient. Recent work of Montejo‐Kovacevich et al. (2019), Montejo‐Kovacevich et al. (2022), Montejo‐Kovacevich, Salazar, et al. (2021) and Meier et al. (2021) showed the effects of elevation gradient in shaping wing morphology and genetic local adaption in *H. erato* and *H. melpomene*. In this context, our study further highlights the role that the environment can play in shaping local populations and contribute to species formation.

## 5 CONCLUSIONS

This is the first study showing that environmental factors can predict large‐scale distributions of *Heliconius* phenotypes. In particular, Neotropical distributions of phenotypes belonging to the red‐yellow Müllerian mimicry are strongly associated with thermal and precipitation variables. These factors are likely to have shared effects on most of the *H. erato* and *H. melpomene* co‐mimics, both via direct and indirect mechanisms. In contrast, local‐scale distributions are more complex to predict, likely due to local effects such as positive frequency‐selection by predators, variable geological histories, and stochastic events. Thus, local phenotype–environment associations can be highly context dependent (see also Boukili & Chazdon, 2017; Sandel & Smith, 2009; Sletvold, 2019). Overall, our results demonstrate the importance of performing multiscale analyses to test hypotheses about the mechanisms involved in the distribution of phenotypes. While large‐scale analyses cannot depict all the intricacies involved in the distribution of phenotypes, they can contribute to our understanding of how large‐scale environmental gradients drive the distribution of species and populations. Therefore, a combination of large and local‐scale analyses may allow a more comprehensive view of the nuanced roles that different environmental factors play in shaping phenotypic distributions across spatial scales and ecological contexts.

## AUTHOR CONTRIBUTIONS

**Ananda R. Pereira Martins:** Conceptualization (equal); data curation (lead); formal analysis (lead); investigation (equal); methodology (equal); project administration (lead); visualization (equal); writing – original draft (equal); writing – review and editing (equal). **Lucas P. Martins:** Conceptualization (supporting); data curation (supporting); formal analysis (equal); methodology (equal); project administration (supporting); writing – original draft (equal); writing – review and editing (equal). **Wing‐Zheng Ho:** Data curation (supporting); formal analysis (equal); methodology (equal); writing – original draft (supporting); writing – review and editing (supporting). **William Owen McMillan:** Conceptualization (equal); data curation (equal); formal analysis (equal); investigation (equal); project administration (equal); resources (lead); supervision (lead); writing – original draft (equal); writing – review and editing (equal). **Jonathan S. Ready:** Conceptualization (equal); data curation (supporting); formal analysis (supporting); methodology (equal); writing – original draft (equal); writing – review and editing (equal). **Rowan Barrett:** Conceptualization (equal); data curation (equal); formal analysis (equal); methodology (equal); project administration (equal); resources (lead); supervision (lead); writing – original draft (equal); writing – review and editing (equal).

## CONFLICT OF INTEREST

The authors declare no competing interests.

## 6 FUNDING INFORMATION

This study was financed by the Coordenação de Aperfeiçoamento de Pessoal de Nível Superior – Brasil (CAPES) – Finance Code 001 (grant 99999.002113/2015‐05), and by the National Sciences and Engineering Research Concil of Canada (NSERC CREATE in Biodiversity, Ecosystem, Services and Sustainability – 466 and NSERC RGPIN 429).

### DATA AVAIBILITY STATEMENT

Data and codes used in this study are archived at: Pereira Martins, Ananda Regina et al. (2022), Scale‐dependent environmental effects on phenotypic distributions in *Heliconius* butterflies, Dryad, Dataset, https://doi.org/10.5061/dryad.j3tx95xh3.

### OPEN RESEARCH BADGES

This article has earned Open Data and Open Materials badges. Data and materials are available at https://doi.org/10.5061/dryad.j3tx95xh3.

## Supporting information


Appendix S1
