# Corrigendum

**DOI:** 10.1002/ece3.9576

**Published:** 2022-12-04

In the recent article by Pereira Martins et al. (2022), the authors would like to add the middle initials of the last author, so it reads as “Rowan D. H. Barrett”.

The authors would also like to add the following ORCIDs for Rowan D. H. Barrett and William Owen McMillan:

Rowan D. H. Barrett: https://orcid.org/0000‐0003‐3044‐2531

William Owen McMillan: https://orcid.org/0000‐0003‐2805‐2745

## References

[ece39576-bib-0001] Pereira Martins, A. R. , Martins, L. P. , Ho, W.‐Z. , McMillan, W. O. , Ready, J. S. , & Barrett, R. (2022). Scale‐dependent environmental effects on phenotypic distributions in *Heliconius* butterflies. Ecology and Evolution, 12, e9286. 10.1002/ece3.9286 36177141 PMC9471044

