# Supplementary material for: Scale‐dependent environmental effects on phenotypic distributions in Heliconius butterflies
Source: Ecol Evol. 2022 Sep 13;12(9):e9286. doi: 10.1002/ece3.9286 (PMC9471044; doi:10.1002/ece3.9286)
Supplement: Supplementary file 1 — Appendix S1 [file ECE3-12-e9286-s001.docx]

Supplementary material

Table 1. Sampling location coordinates across the Brazilian Amazon where we collected *Heliconius erato* and *H. melpomene*.

| Location ID | Location name | Brazilian State | Transect | Longitude | Latitude |
| --- | --- | --- | --- | --- | --- |
| CAC | Cachoeira do Arari | Pará | 1 | -48.946833 | -1.033361 |
| VDP | Vila Deus Proverá – Cachoeira do Arari | Pará | 1 | -48.735361 | -1.021444 |
| VN | Vila Nova – Cachoeira do Arari | Pará | 1 | -48.729139 | -1.0087778 |
| COND | Condeixa – Salvaterra | Pará | 1 | -48.5945 | -0.9042222 |
| SOU | Soure | Pará | 1 | -48.51725 | -0.6819167 |
| BAR | Barcarena | Pará | 1 | -48.491472 | -1.6999167 |
| UTI | Parque Estadual do Utinga – Belém | Pará | 1 | -48.439 | -1.4246944 |
| BENE | Benevides | Pará | 1 | -48.260222 | -1.3519167 |
| ISA | Santa Isabel do Pará | Pará | 1 | -48.036528 | -1.3078611 |
| CAST | Castanhal | Pará | 1 | -47.826278 | -1.3033611 |
| STA.MA | Ramal Miritueira – Santa Maria do Pará | Pará | 1 | -47.483583 | -1.3286667 |
| CAPA | Capanema | Pará | 1 | -47.249694 | -1.2525833 |
| MURU | Muruteua – Santa Luzia do Pará | Pará | 1 | -47.004361 | -1.39225 |
| TRA | Rio das Pedras – Tracuateua | Pará | 1 | -46.943028 | -1.1436111 |
| STOANT | Santo Antônio – Bragança | Pará | 1 | -46.865694 | -1.5448333 |
| BRA | Bragança | Pará | 1 | -46.739183 | -1.077283 |
| NOVA | Fazenda Nova Aliança – Viseu | Pará | 1 | -46.630667 | -1.7041667 |
| Cpi | Cachoeira do Piriá | Pará | 1 | -46.420806 | -1.7756667 |
| BVG | Boa Vista do Gurupi | Maranhão | 1 | -46.163389 | -1.7922778 |
| 7IR | Fazenda Sete Irmãos - Cândido Mendes | Maranhão | 1 | -45.788528 | -1.8604722 |
| BG | Baixo Grande – Turilândia | Maranhão | 1 | -45.336722 | -2.1270556 |
| MOC | Mocambo – Santa Helena | Maranhão | 1 | -45.179861 | -2.3156389 |
| MAL | Malhado – Pinheiro | Maranhão | 1 | -45.003028 | -2.5378611 |
| BURIT | Fazenda Buritirana – Peri-Merim | Maranhão | 1 | -44.847667 | -2.639694 |
| CENT | Centrinho – Bequimão | Maranhão | 1 | -44.74266 | -2.4510278 |
| SANT | Santana – Bequimão | Maranhão | 1 | -44.704083 | -2.4468889 |
| ZP | Zé Pedro – Bacabeira | Maranhão | 1 | -44.350778 | -2.9943333 |
| CUJ | Cujupe – Alcântara | Maranhão | 1 | -44.550277 | -2.4860278 |
| IG | Igaraú – São Luís | Maranhão | 1 | -44.324028 | -2.7564722 |
| MRC | Maracanã – São Luís | Maranhão | 1 | -44.28392 | -2.61401 |
| ITA | Reserva do Itapiracó – São Luís | Maranhão | 1 | -44.214111 | -2.5285 |
| RAP | Raposa | Maranhão | 1 | -44.154527 | -2.4596667 |
| SAG | Sítio Aguahy – São José de Ribamar | Maranhão | 1 | -44.149194 | -2.6491389 |
| AX | Axixá | Maranhão | 1 | -44.100694 | -2.8458889 |
| IC | Icatu | Maranhão | 1 | -44.037167 | -2.8045278 |
| BOQ | Boqueirão – Icatu | Maranhão | 1 | -43.820889 | -2.8055833 |
| PR | Prata – Humberto de Campos | Maranhão | 1 | -43.548556 | -2.7428056 |
| ALGODAO | Algodão – Primeira Cruz | Maranhão | 1 | -43.314608 | -2.785167 |
| HC | Humberto de Campos | Maranhão | 1 | -43.456111 | -2.615 |
| PEDRAS | Pedras – Santo Amaro | Maranhão | 1 | -43.075103 | -2.834036 |
| ABAE | Abaetetuba | Pará | 2 | -48.8110555 | - 1.7538889 |
| MUCA | Mucajeteua - Igarapé Mirim | Pará | 2 | -48.8919722 | - 1.9755278 |
| IGM | Igarapé Mirim | Pará | 2 | -49.1218611 | -2.1571944 |
| CARAP | Carapajó - Cametá | Pará | 2 | -49.3181111 | -2.3071944 |
| ARE | Areião – Cametá | Pará | 2 | -49.4172777 | -2.5068611 |
| MOCA | Mocajuba | Pará | 2 | -49.5536111 | -2.6666944 |
| ANAN02 | PA-151 - Cardoso | Pará | 2 | -49.62891 | -2.94488 |
| CARD | Cardoso - Baião | Pará | 2 | -49.65767 | -2.90814 |
| CAM | Cametá | Pará | 2 | -49.5155 | -2.1898056 |
| UXI | Uxi – Oeiras do Pará | Pará | 2 | -49.735917 | -2.3039444 |
| FR | França – Oeiras do Pará | Pará | 2 | -49.783306 | -2.6242778 |
| PI | Piçarreira – Cametá | Pará | 2 | -49.708861 | -2.285472 |
| SDP | Deus Proverá – Baião Km140 | Pará | 2 | -49.779389 | -2.6531111 |
| ALE2.2 | Bom Cuidado – Alenquer | Pará | 3 | -54.663556 | -1.5875 |
| ALE2.1 | Bom Cuidado – Alenquer | Pará | 3 | -54.696139 | -1.589861 |
| ALE | Perpétuo Socorro - Alenquer | Pará | 3 | -54.663556 | -1.5875 |
| OX | Oriximiná | Pará | 3 | -55.803033 | -1.697233 |
| SAN1 | Santarém | Pará | 3 | -54.788283 | -2.45515 |
| SAN2 | Santarém | Pará | 3 | -54.791233 | -2.45585 |
| AL | Alter do Chão – Santarém | Pará | 3 | -54.95915 | -2.485033 |
| BEL | Belterra | Pará | 3 | -54.916889 | -2.732472 |
| TAPA1 | Flona Tapajós | Pará | 3 | -54.935889 | -2.935639 |
| TAPA2 | Flona Tapajós | Pará | 3 | -54.954639 | -3.358444 |
| TAPA3 | Flona Tapajós | Pará | 3 | -54.825861 | -3.793556 |
| TAPA4 | Flona Tapajós | Pará | 3 | -54.937194 | -4.095639 |
| IRAN | Iranduba | Amazonas | 4 | -60.140194 | -3.148806 |
| BOA | Boa Vista - Manacapuru | Amazonas | 4 | -60.451583 | -3.296972 |
| CIR | Cirandeira Bela - Manacapuru | Amazonas | 4 | -60.451583 | -3.296972 |
| ANA | Anavilhanas Hotel – Novo Airão | Amazonas | 4 | -60.925806 | -2.638444 |
| RDS | Rio Negro - Iranduba | Amazonas | 4 | -60.740917 | -3.069833 |
| IRAN | Iranduba | Amazonas | 4 | -60.155167 | -3.151694 |
| MAO | Ramal das Castanheiras - Manaus | Amazonas | 4 | -60.010278 | -2.827944 |
| PRES | Presidente Figueiredo | Amazonas | 4 | -60.037472 | -2.392028 |
| MA | Ramal Maranhão – Pres. Figueiredo | Amazonas | 4 | -60.137194 | -1.732389 |
| PAUL | Ramal do Paulista – Pres. Figueiredo | Amazonas | 4 | -60.268056 | -1.467639 |
| CIRA | Cachoeira Iracema– Pres. Figueiredo | Amazonas | 4 | -60.061194 | -1.985222 |
| ABO | Abonari – Pres. Figueiredo | Amazonas | 4 | -60.344556 | -1.276472 |


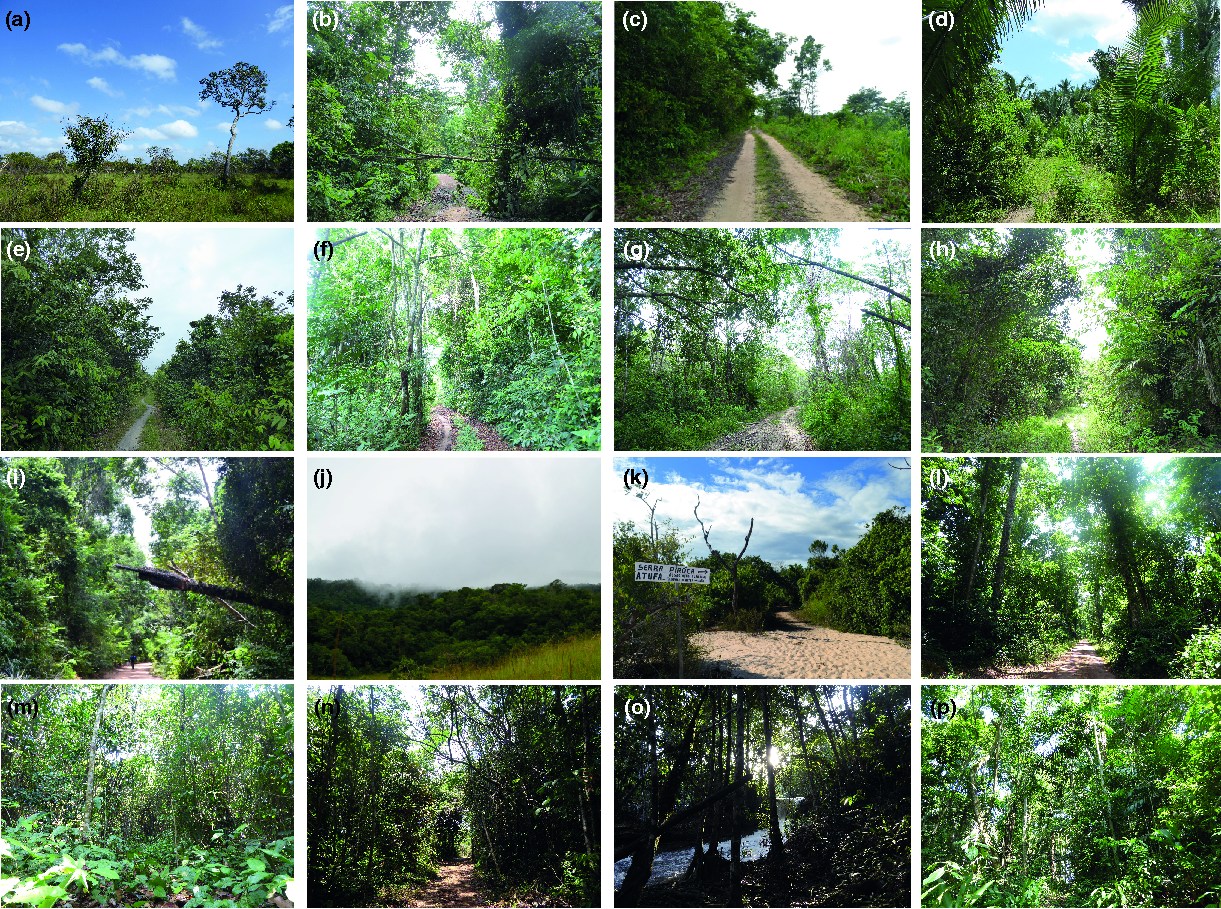


Figure 1. Examples of sampling sites. (a-d) Transect 1. (a) Cachoeira do Arari (CAC). (b) Utinga (UTI). (c) Fazenda Sete Irmãos (7IR – site within hybrid zone). (d) Baixo Grande (BG – site within hybrid zone). (e-h) Transect 2. (e) Carapajó (CARAP). (f) Cardoso (CARD). (g) Cametá (CAM). (h) Só Deus Proverá (SDP). (i-l) Transect 3. (i) Oriximiná (OX – site within hybrid zone). (j) Santarém (SAN1 – site within hybrid zone). (k) Alter do Chão (AL – site within hybrid zone). (l) Tapajós (TAPA1). (m-p) Transect 4. (m) Boa Vista (BOA). (n) Iranduba (IRAN). (o) Cachoeira Iracema (CIRA). (p) Ramal Maranhão (MA).

Table 2. *Heliconius erato* and *H. melpomene* races used in the phenotypic distribution modelling. For each species, races belonging to the same phenotype were considered as a single group to produce a predicted distribution map. 94% of the occurrence points are available in Rosser et al. 2012 (https://heliconius-maps.github.io/) and 6% are from our own fieldwork.

| ***Heliconius erato*** | ***Heliconius melpomene*** | **Phenotypes** | **Number of Samples**  ***H. erato* \| *H. melpomene*** |
| --- | --- | --- | --- |
| *H. e. hydara* | *H. m. melpomene* | Postman | 444 \| 362 |
| *H. e. magnifica* | *H. m. anduzei* |  |  |
| *H. e. guarica* | *H. m. euryades* |  |  |
| *H. e. amphitrite* | *H. m. flagrans* |  |  |
| *H. e. adana* | *H. m. pyrforus* |  |  |
| *H. e. tobagoensis* | *H. m. tessa* |  |  |
| *H. e. lichyi* | *H. m. vulcanus* |  |  |
| *H. e. phyllis* | *H. m. burchelli* | Postman with hindwing yellow bar | 1038 \| 369 |
| *H. e. favorinus* | *H. m. nanna* |  |  |
| *H. e. petiverana* | *H. m. amandus* |  |  |
| *H. e. demophoon* | *H. m. amaryllis* |  |  |
| *H. e. dignus* | *H. m. bellula* |  |  |
| *H. e. colombina* | *H. m. rosina* |  |  |
| *H. e. cruentus* |  |  |  |
| *H. e. amalfreda* | *H. m. meriana* | Dennis | 70 \| 32 |
| *H. e. amazona* | *H. m. thelxiope* | Dennis-ray | 668 \| 420 |
| *H. e. erato* | *H. m. madeira* |  |  |
| *H. e. emma* | *H. m. aglaope* |  |  |
| *H. e. estrella* | *H. m. intersectus* |  |  |
| *H. e. etylus* | *H. m. ecuadorensis* |  |  |
| *H. e. lativitta* | *H. m. malleti* |  |  |
| *H. e. luscombei* | *H. m. michellae* |  |  |
| *H. e. reductimacula* | *H. m. penelope* |  |  |
| *H. e. venustus* | *H. m. schunkei* |  |  |
|  | *H. m. thelxiopeia* |  |  |
|  | *H. m. vicina* |  |  |


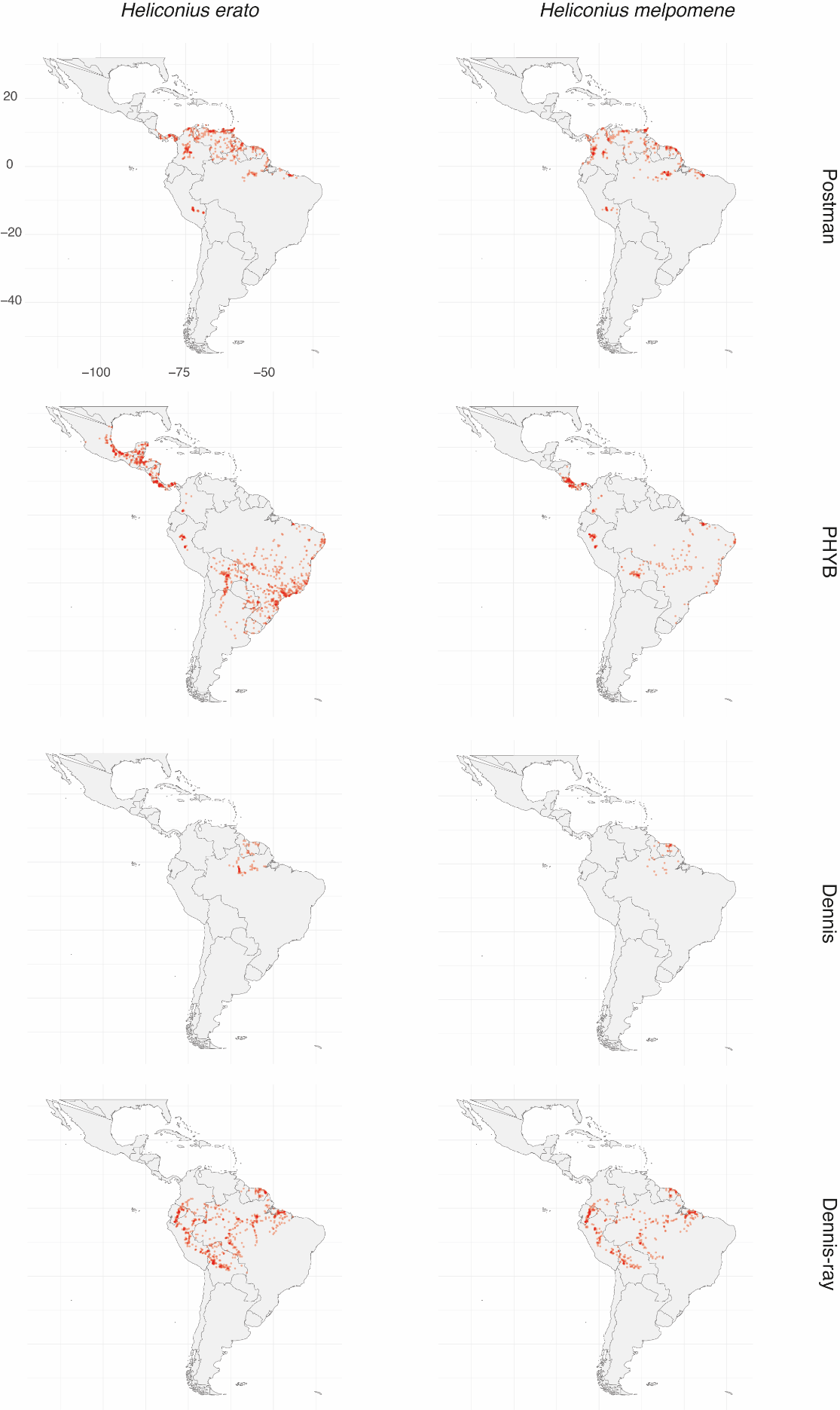


Figure 2. Maps with *Heliconius erato* and *H. melpomene* phenotypic occurrence points.

Table 3. Environmental variables used in the phenotypic distribution modelling (large-scale analysis) and in local association analysis.

Variables used as predictors in the phenotypic distribution modelling after filtering based on Pearson’s correlation (r < 0.7) are highlighted in bold font. Variables used in the local-scale association analysis are indicated by a red dot.

| **Environmental variables** | **Class** | **Data base** | **Original spatial resolution** | **Potential effects on *Heliconius*** |
| --- | --- | --- | --- | --- |
| **1. Mean annual air temperature –** **°C** | Temperature | CHELSA  (<https://chelsa-climate.org>)  (Karger et al. 2017) | 1km | Butterfly physiology, population dynamics, life-history traits, and food resources (Dobkin et al. 1987, Hanspach et al. 2014, Dilts et al. 2019). |
| 2. Mean diurnal range |  |  |  |  |
| 3. Isothermality |  |  |  |  |
| **4. * Temperature seasonality – °C (standard deviation * 100)** |  |  |  |  |
| 5. Maximum temperature of warmest month |  |  |  |  |
| 6. Minimum temperature of coldest month |  |  |  |  |
| 7. Temperature annual range |  |  |  |  |
| 8. Mean temperature of wettest quarter |  |  |  |  |
| 9. Mean temperature of driest quarter |  |  |  |  |
| 10. Mean temperature of warmest quarter |  |  |  |  |
| 11.Mean temperature of coldest quarter |  |  |  |  |
| **12. Annual precipitation – mm/year** | Precipitation |  |  |  |
| 13. Precipitation of wettest month |  |  |  |  |
| 14. Precipitation of driest month |  |  |  |  |
| **15. ** Precipitation seasonality – mm (standard deviation * 100)** |  |  |  |  |
| 16. Precipitation of wettest quarter |  |  |  |  |
| 17. Precipitation of driest quarter |  |  |  |  |
| **18. Precipitation of warmest quarter (mean) – mm** |  |  |  |  |
| 19. Precipitation of coldest quarter |  |  |  |  |
|  | Soil | Soil Grids (<https://soilgrids.org>) | 250m | Quality and availability of host plants (Benson et al. 1975, Kerpel et al. 2006, Krämer et al. 2012). |
| 20. pH |  |  |  |  |
| **21. Cation exchange capacity (CEC) – cmol(c)/kg** |  |  |  |  |
| **22. Soil organic carbon content (SOC) – g per kg** |  |  |  |  |
| **23. Clay content (0–2 micrometer) –** **g/100g** |  |  |  |  |
| **24. Sand content (50–2000 micrometer) –** **g/100g** |  |  |  |  |
| 25. Silt content (2–50 micrometer) |  |  |  |  |
| 26. *** Normalized difference vegetation index (NDVI) V.2 | Vegetation | Copernicus Global Land Service  (<https://land.copernicus.vgt.vito.be/>) | 300m | Quality and availability of host plants and butterfly dispersal (Benson et al. 1975, Kerpel et al. 2006, DeVries et al. 2010, Cormont et al. 2011, Krämer et al. 2012, Kuussaari et al. 2016, Knight et al. 2019). |
| **27. Percentage tree cover – %** |  | Global Mapping Project (<https://globalmaps.github.io>) | 1km |  |
| 28. Forest canopy height | Land | Global Land Analysis & Discovery (<https://glad.umd.edu/dataset/gedi>) | 30m | Butterfly dispersal (DeVries et al. 2010, Cormont et al. 2011, Kuussaari et al. 2016, Knight et al. 2019). |
| 29. Terrain elevation above the sea level - meters |  | NASA visible earth  (<https://globalsolaratlas.info>) | 1km |  |
| **30. Wind speed – meters/second** | Wind | Global Wind Atlas  (<https://globalwindatlas.info>) | 250m | Butterfly dispersal (DeVries et al. 2010, Cormont et al. 2011, Kuussaari et al. 2016, Knight et al. 2019). |
| **31. Global horizontal irradiation (annual average) – kWh/m^2^** | Solar data | Global Solar Atlas  (<https://globalsolaratlas.info>) | 250m | Butterfly physiology, population dynamics, life-history traits, and food resources (Dobkin et al. 1987, Hanspach et al. 2014, Dilts et al. 2019). |

***** Temperature seasonality is a measure of temperature change over the year, calculated as the standard deviation of monthly temperature averages throughout 1979-2013. The result is multiplied by 100, which was designed to preserve significant digits (O’Donnell and Ignizio, D. A. 2012, Karger et al. 2019).

** Precipitation seasonality is a measure of the variation in monthly precipitation totals over the course of the year. This index is the ratio of the standard deviation of the monthly total precipitation to the mean monthly total precipitation (also known as the coefficient of variation) (O’Donnell and Ignizio, D. A. 2012, Karger et al. 2019).

*** Normalized Difference Vegetation Index (NDVI) measures the density of vegetation of a region, varying from -0.08, indicating no vegetation, to 0.92, indicating dense vegetation (Swinnen et al. 2021).

Table 4. Maxent tuned model parameters.

| **Phenotypes** | Feature class combinations (fc: linear, quadratic, product, threshold and hinge). Default = lqph | Regularization multiplier. Default = 1 | Number of iterations used by the algorithm. Default = 500 |
| --- | --- | --- | --- |
|  | lqpht  lqph | 2.1  1.9 | 921  921 |
| **Postman** |  |  |  |
| *H. erato*  *H. melpomene* |  |  |  |
|  |  |  |  |
|  | lqph  lqpht | 8.3  5.7 | 131  91 |
| **Postman-HYB** |  |  |  |
| *H. erato*  *H. melpomene* |  |  |  |
|  |  |  |  |
|  | lqph  lqph | 3.5  0.8 | 201  271 |
| **Dennis** |  |  |  |
| *H. erato*  *H. melpomene* |  |  |  |
|  | lh  lqpht | 1  8.3 | 181  621 |
| **Dennis-Ray** |  |  |  |
| *H. erato*  *H. melpomene* |  |  |  |
|  |  |  |  |

Table 5. Races of *Heliconius erato* and *Heliconius melpomene* used in color variation analyses.

| ***Heliconius erato*** | ***Heliconius melpomene*** | **Number of Samples**  ***H. erato* \| *H. melpomene*** | **Phenotypes** |
| --- | --- | --- | --- |
| *H. e. hydara* | *H. m. melpomene* | 41 \| 110 | Postman |
| *H. e. phyllis* | *H. m. burchelli/H. m. nanna* | 23 \| 43 | Postman with hindwing yellow bar |
| *H. e. amalfreda* | *H. m. meriana* | 36 \| 4 | Dennis |
| *H. e. amazona* | *H. m. thelxiope* | 146 \| 39 | Dennis-ray |
| *H. e. erato* | *H. m. madeira* | 2 \| 28 |  |
| *H. e. estrella* | *H. m. intersectus* | 7 \| 17 |  |
| *H. e. lativitta* |  | 8 |  |
| *H. e. reductimacula* |  | 7 |  |

**(b)**

**(a)**


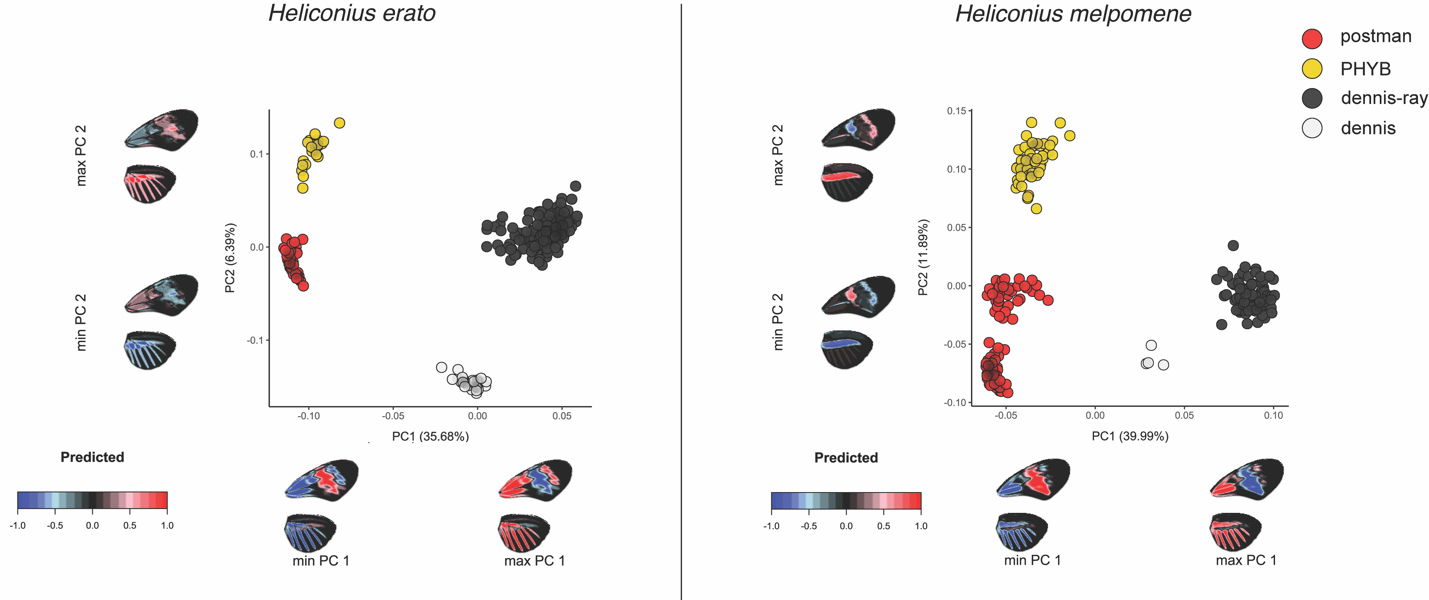


**(c)**

**(d)**


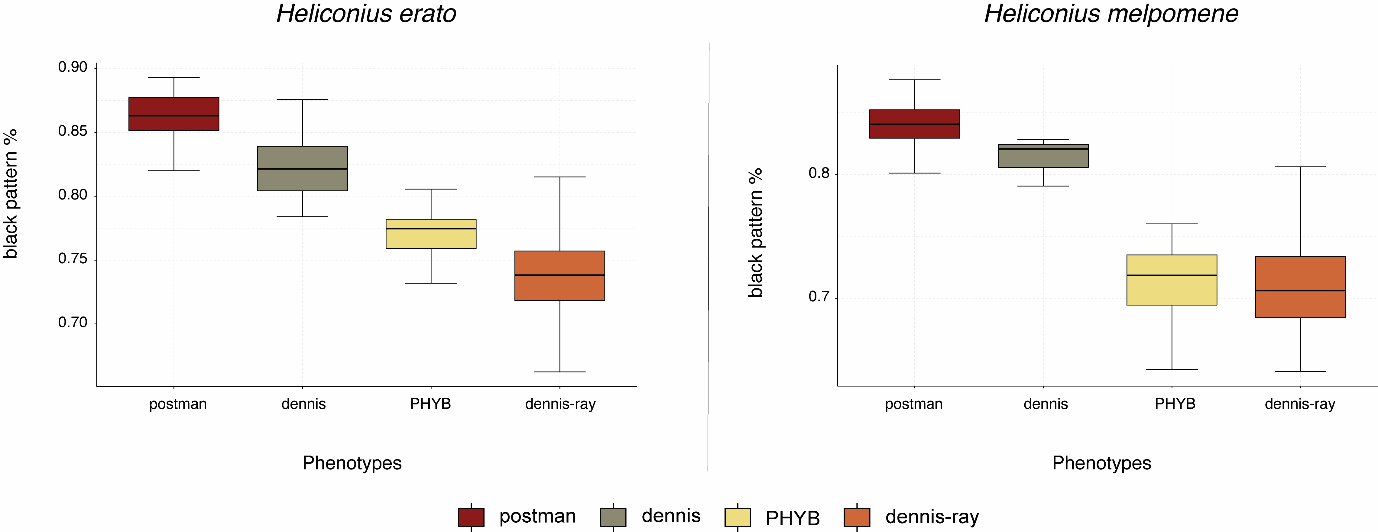


Figure 3. Color variation and black pattern analyses. (a) *Heliconius erato* color variation PCA. (b) *Heliconius melpomene* color variation PCA. Wing predicted patterns vary from absence (-1: blue) to presence (1: red). (c) *H. erato* wing black pattern comparison (ANOVA F = 268.7, p < 0.001). Postman, dennis, PHYB and dennis-ray form statistically significant different melanic morphs (Tukey: p < 0.001 for all the possible pair-wise comparisons). (d) *H. melpomene* wing black pattern comparison (ANOVA F = 427.6, p < 0.001). Postman and dennis phenotypes are not statistically significant distinct morphs in terms of black patterning, as well as PHYB and dennis-ray. However, the two phenotypes with higher melanization (postman + dennis) are different from the two phenotypes with lower melanization (PHYB + dennis-ray) (Tukey: p < 0.001).

Table 6. Models’ evaluation metrics: Area Under the Curve of the Receiver Operating Characteristic (AUC) and the True Skill Statistic (TSS). (10 repetitions per model: 10-fold cross validation).

| **Phenotypes** | Repetitions | Postman  Model 1 | PHYB  Model 2 | Dennis  Model 3 | Dennis-ray  Model 4 |  | Postman  Model 5 | PHYB  Model 6 | Dennis  Model 7 | Dennis-ray  Model 8 |
| --- | --- | --- | --- | --- | --- | --- | --- | --- | --- | --- |
|  |  | H*. erato* | | | |  | *H. melpomene* | | | |
| **AUC**  **train** | 1  2  3  4  5  6  7  8  9  10 | 0.9435  0.9408  0.9413  0.9444  0.9427  0.9429  0.9421  0.9415  0.9434  0.9435 | 0.8539  0.8558  0.8580  0.8540  0.8525  0.8539  0.8532  0.8516  0.8512  0.8555 | 0.9698  0.9684  0.9679  0.9731  0.9694  0.9725  0.9668  0.9717  0.9717  0.9691 | 0.9004  0.8980  0.8957  0.9019  0.9000  0.9030  0.8988  0.8946  0.8997  0.9007 |  | 0.9360  0.9394  0.9363  0.9393  0.9394  0.9371  0.9419  0.9391  0.9402  0.9415 | 0.8793  0.8762  0.8756  0.8771  0.8879  0.8878  0.8772  0.8848  0.8698  0.8820 | 0.9734  0.9806  0.9754  0.9811  0.9793  0.9807  0.9764  0.9766  0.9773  0.9759 | 0.8655  0.8658  0.8663  0.8647  0.8715  0.8619  0.8650  0.8680  0.8692  0.8677 |
| **AUC**  **test** | 1  2  3  4  5  6  7  8  9  10 | 0.9251  0.9256  0.9276  0.9241  0.9244  0.9246  0.9248  0.9254  0.9253  0.9260 | 0.8519  0.8535  0.8543  0.8496  0.8502  0.8523  0.8504  0.8518  0.8487  0.8535 | 0.9757  0.9784  0.9757  0.9766  0.9795  0.9780  0.9766  0.9781  0.9719  0.9764 | 0.8625  0.8608  0.8601  0.8621  0.8640  0.8619  0.8649  0.8615  0.8670  0.8694 |  | 0.9431  0.9434  0.9442  0.9421  0.9402  0.9456  0.9454  0.9422  0.9441  0.9446 | 0.8607  0.8432  0.8645  0.8639  0.8556  0.8619  0.8662  0.8622  0.8568  0.8553 | 0.9681  0.9666  0.9655  0.9665  0.9693  0.9684  0.9694  0.9658  0.9719  0.9652 | 0.8729  0.8711  0.8698  0.8738  0.8734  0.8733  0.8711  0.8711  0.8749  0.8724 |
| **TSS**  **train** | 1  2  3  4  5  6  7  8  9  10 | 0.7486  0.7413  0.7411  0.7469  0.7450  0.7469  0.7351  0.7500  0.7543  0.7364 | 0.5424  0.5461  0.5465  0.5385  0.5331  0.5395  0.5374  0.5251  0.5261  0.5383 | 0.8374  0.8474  0.8298  0.8883  0.8590  0.8637  0.8461  0.8897  0.8695  0.8713 | 0.6315  0.6317  0.6221  0.6431  0.6449  0.6446  0.6292  0.6240  0.6372  0.6370 |  | 0.7436  0.7517  0.7400  0.7434  0.7582  0.7476  0.7459  0.7537  0.7484  0.7524 | 0.5972  0.5898  0.5915  0.6030  0.6098  0.6167  0.5989  0.6056  0.5740  0.6079 | 0.8572  0.8879  0.8601  0.8887  0.8843  0.8840  0.8507  0.8648  0.8626  0.8694 | 0.5602  0.5629  0.5634  0.5625  0.5689  0.5564  0.5554  0.5688  0.5642  0.5622 |
| **TSS**  **test** | 1  2  3  4  5  6  7  8  9  10 | 0.7473  0.7339  0.7427  0.7352  0.7213  0.7243  0.7459  0.7280  0.7308  0.7471 | 0.5315  0.5408  0.5373  0.5394  0.5367  0.5369  0.5333  0.5380  0.5234  0.5340 | 0.8543  0.8831  0.8501  0.9045  0.8894  0.8848  0.8831  0.8790  0.8622  0.8831 | 0.5816  0.5801  0.5876  0.5824  0.5859  0.5762  0.5889  0.5832  0.5818  0.5988 |  | 0.7425  0.7536  0.7528  0.7526  0.7344  0.7698  0.7529  0.7439  0.7363  0.7466 | 0.5636  0.5380  0.5938  0.5950  0.5765  0.5751  0.5726  0.5804  0.5742  0.5764 | 0.9335  0.8771  0.9219  0.9178  0.9103  0.9381  0.9236  0.9269  0.9273  0.8904 | 0.5637  0.5566  0.5616  0.5645  0.5783  0.5759  0.5514  0.5605  0.5745  0.5562 |


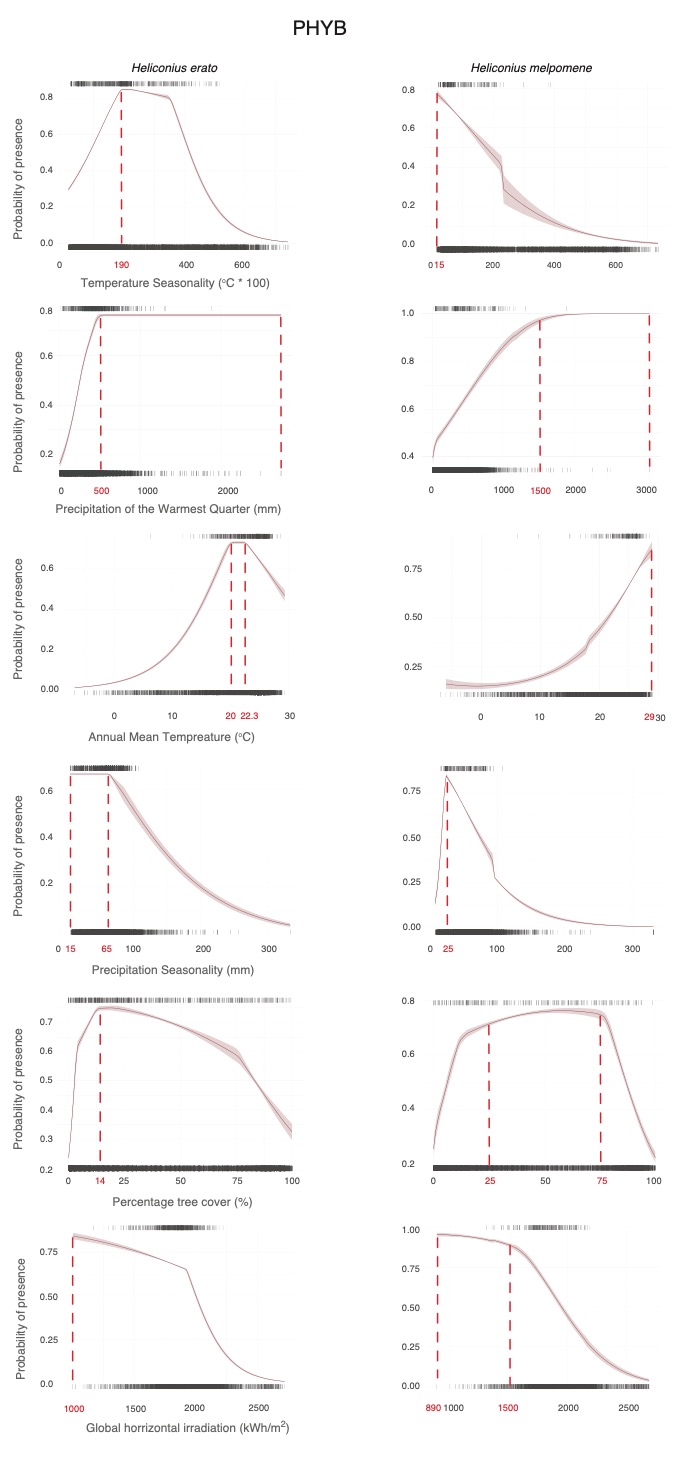


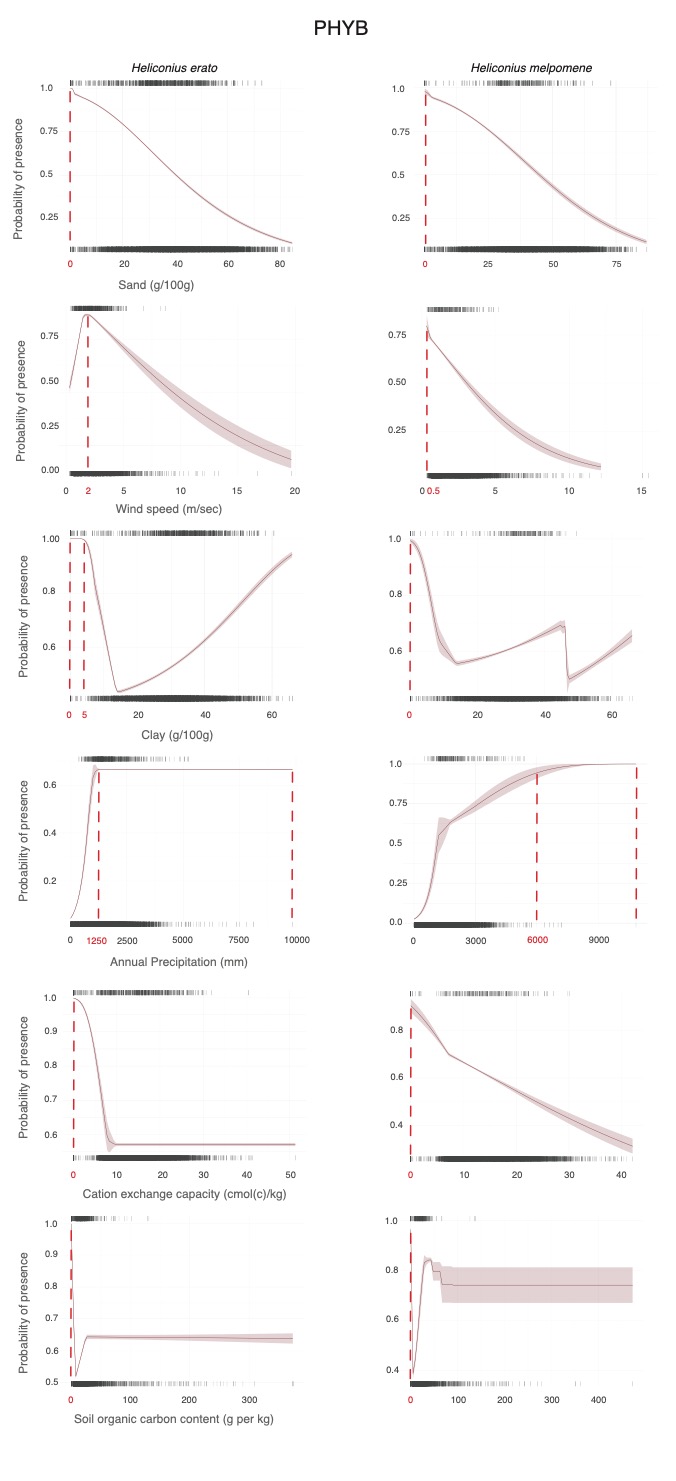


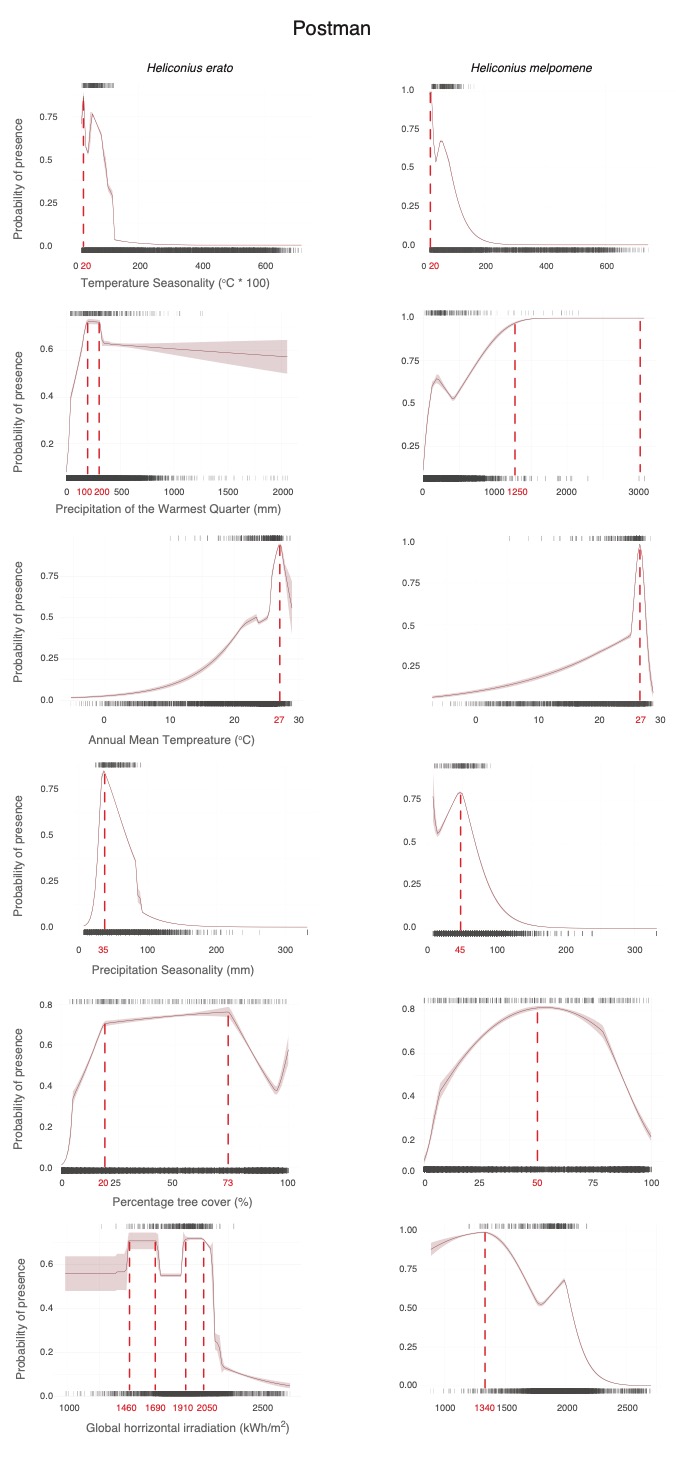


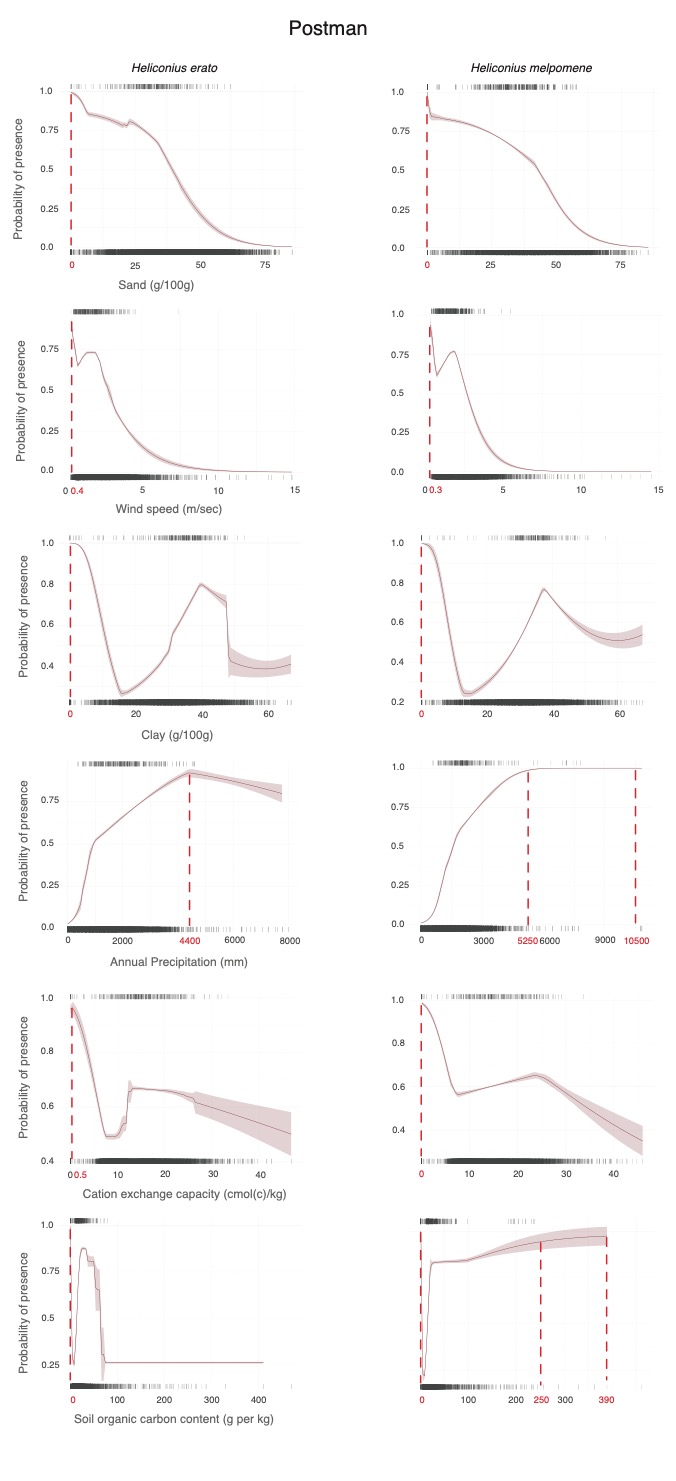


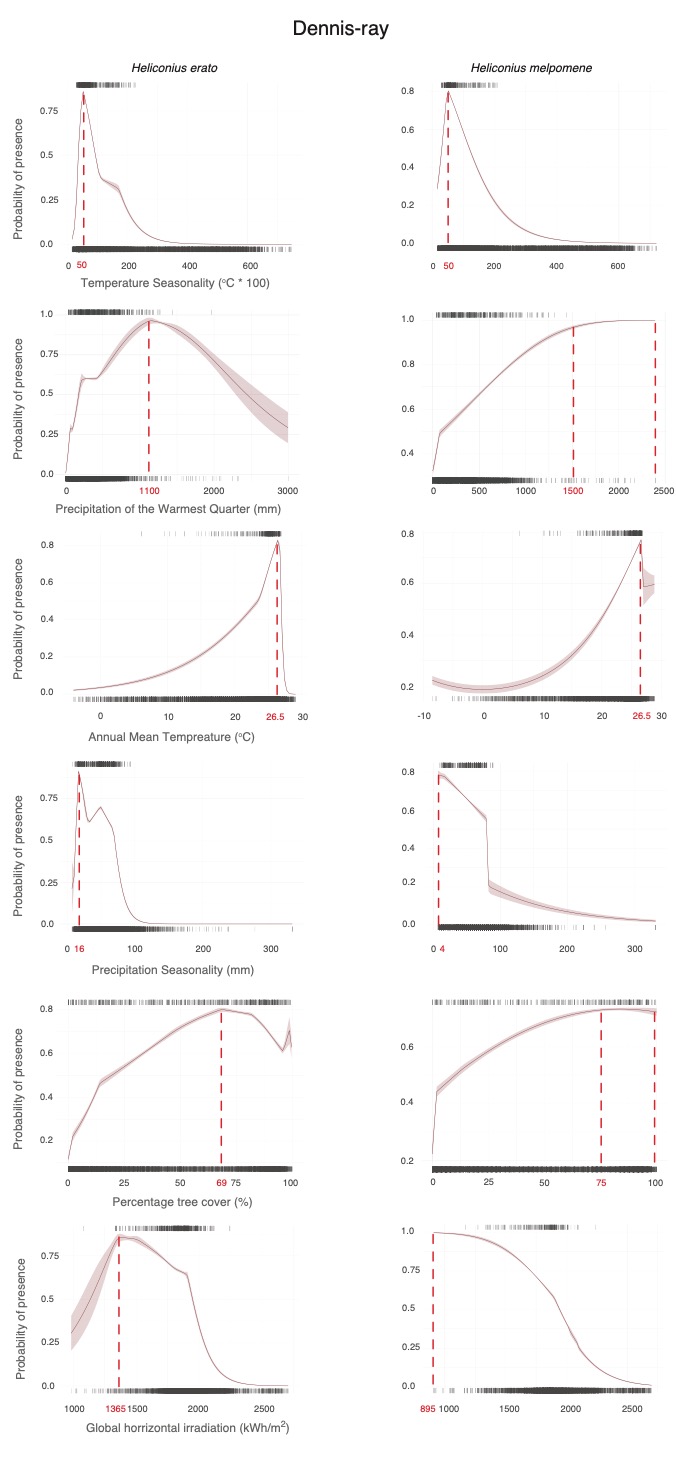


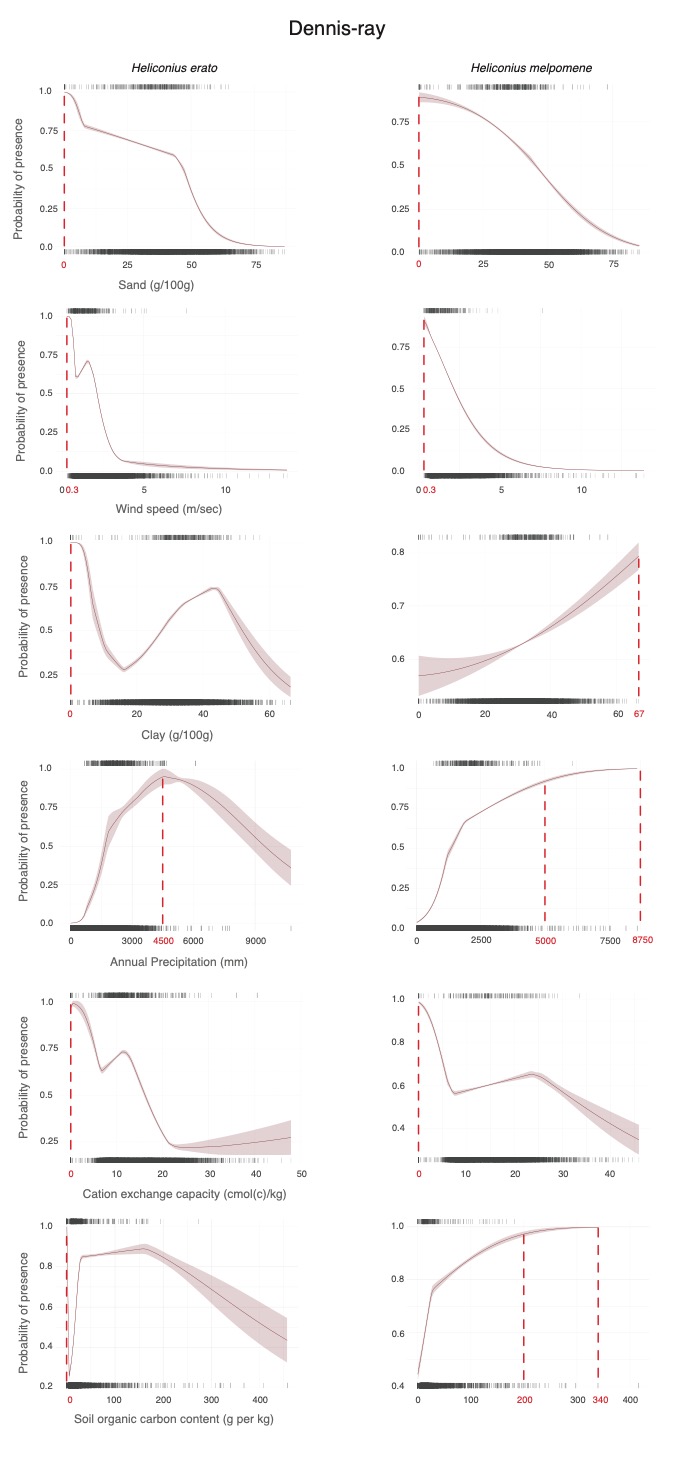


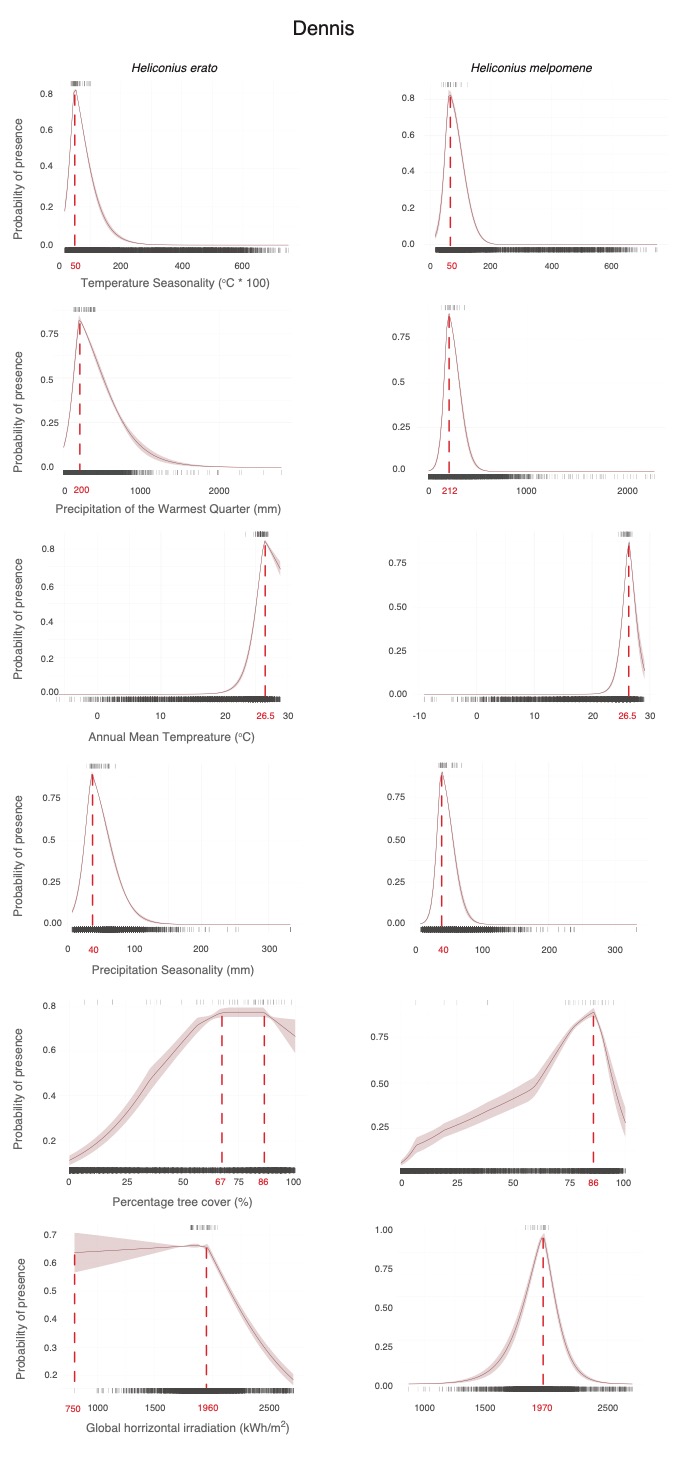


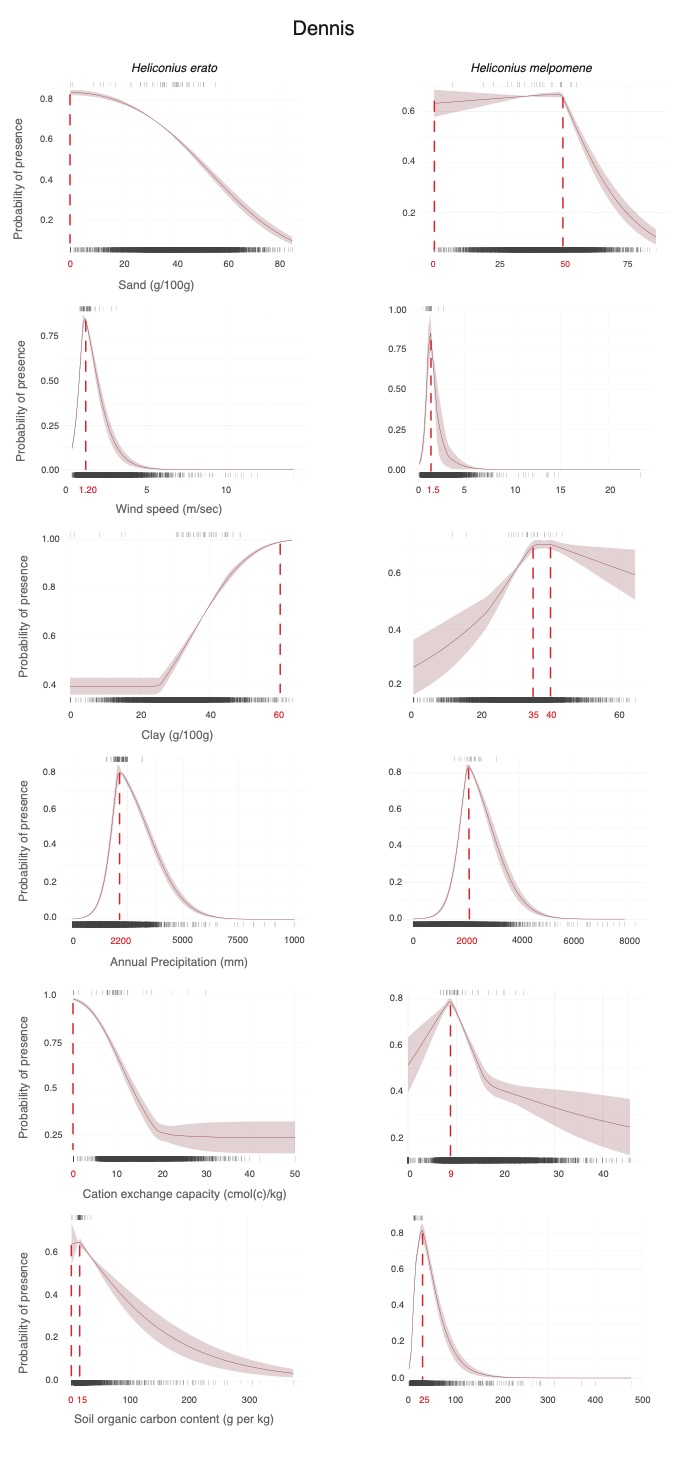


Figure 4. Logistic Maxent response curves showing how the presence’s probability of *H. erato* and *H. melpomene* phenotypes vary along environmental gradients. Environmental optima values associated with higher probability of presence are highlighted in dashed red lines. Response curves plots use 95% confidence interval.

Table 7. Mean parameter estimates with 95% of confidence interval for each cline model. *Heliconius melpomene* presents two sets of parameters because cline analyses were performed based on the westernmost and northmost site within the transect and based on the westernmost and northmost site shared with *H. erato*.

|  | Centre (Km) | Width (Km) | Minimum frequency | Maximum frequency |
| --- | --- | --- | --- | --- |
| *Heliconius erato* | | | | |
| Cline 1: dennis-ray to postman | 310.78 (282.48, 341.44) | 173.68 (122.76, 221.56) | 0.01 (0.00, 0.03) | 0.98 (0.95, 1.00) |
|  |  |  |  |  |
| Cline 2: Absence HYB to presence of HYB | 208.10 (169.46, 249.59) | 194.08 (120.72, 272.47) | 0.05 (0.00, 0.13) | 0.89 (0.80, 0.99) |
|  |  |  |  |  |
| Cline 3: Postman to dennis-ray | 29.64 (-7.78, 65.43) | 95.21 (17.95, 169.94) | 0.10 (0.02, 0.17) | 0.89 (0.80, 0.98) |
|  |  |  |  |  |
| *Heliconius melpomene* | | | | |
| Cline 1: dennis-ray to postman | 355.68 (342.46, 368.12) | 93.74 (61.31, 127.61) | 0.01 (0.00, 0.03) | 1.00 (0.99, 1.00) |
|  |  |  |  |  |
| Cline 2: Absence HYB to presence of HYB | 142.65 (119.82, 163.38) | 149.13 (87.33, 212.31) | 0.10 (0.00, 0.18) | 0.93 (0.84, 1.00) |
|  |  |  |  |  |
| Cline 3: Postman to dennis-ray | 169.37 (132.93, 213.64) | 132.53 (26.98, 232.28) | 0.10 (0.00, 0.19) | 0.93 (0.88, 1.00) |
|  |  |  |  |  |
| *Heliconius melpomene (shared westernmost or northmost site with H. erato)* | | | | |
| Cline 1: dennis-ray to postman | 331.07 (317.56, 343.46) | 92.66 (60.92, 126.27) | 0.01 (0.00, 0.03) | 1.00 (0.99, 1.00) |
|  |  |  |  |  |
| Cline 2: Absence HYB to presence of HYB | 102.07 (82.20, 121.59) | 144.32 (91.91, 209.43) | 0.12 (0.03, 0.20) | 0.96 (0.87, 1.00) |
|  |  |  |  |  |
| Cline 3: Postman to dennis-ray | 169.37 (132.93, 213.64) | 132.53 (26.98, 232.28) | 0.10 (0.00, 0.19) | 0.93 (0.88, 1.00) |

Figure 5. *Heliconius melpomene* local-scale cline analyses using the common westernmost site with *H. erato*. In red: postman frequency change along the transition dennis-ray to postman in Eastern Amazon. In yellow: hindwing yellow bar frequency change along the transition postman to postman with hindwing yellow bar (PHYB) in Eastern Amazon.

Table 8. Spearman correlations between postman frequencies and environmental variables in *H. erato* and *H. melpomene* clines 1 and 3, and between hindwing yellow bar frequencies and environmental variables in cline 2.

|  | Annual mean temperature | Temperature seasonality | Annual precipitation | Precipitation seasonality | GHI | NDVI | % Tree cover |
| --- | --- | --- | --- | --- | --- | --- | --- |
|  |  |  |  |  |  |  |  |
| *Heliconius erato* | | | | | | | |
| Cline 1: dennis-ray to postman  p- value | 0.656  *** | - 0.156  ‘ ’ | - 0.763  *** | 0.761  *** | 0.371  ‘.’ | - 0.065  ‘.’ | 0.505  ‘.’ |
| Cline 2: Absence HYB to presence of HYB  p-value | 0.682  ‘.’ | 0.663  ‘.’ | - 0.444  ‘ ’ | 0.675  ‘.’ | - 0.213  ‘ ’ | - 0.0313  ‘ ’ | - 0.632  ‘ ’ |
| Cline 3: Postman to dennis-ray  p-value | 0.687  * | 0.546  * | - 0.689  ‘ ’ | 0.0723  ‘ ’ | 0.689  * | 0.529  ‘ ’ | 0.42  ‘ ’ |
| *Heliconius melpomene* | | | | | | | |
| Cline 1: dennis-ray to postman  p-value | 0.604  *** | - 0.414  ‘ ’ | - 0.869  *** | 0.865  *** | 0.32  * | - 0.279  ‘ ’ | - 0.0435  ‘ ’ |
| Cline 2: Absence HYB to presence of HYB  p-value | 0.809  *** | 0.79  *** | - 0.0939  ‘ ’ | 0.828  ** | 0.293  ‘ ’ | - 0.0776  ‘ ’ | - 0.359  ‘ ’ |
| Cline 3: Postman to dennis-ray  p-value | - 0.339  ‘ ’ | -0.25  ‘ ’ | 0.162  ‘ ’ | - 0.514  ‘ ’ | - 0.0735  ‘ ’ | - 0.132  ‘ ’ | - 0.485  ‘ ’ |
|  |  |  |  |  |  |  |  |

Signif. codes: 0 ‘***’ 0.001 ‘**’ 0.01 ‘*’ 0.05 ‘.’ 0.1 ‘ ’ 1

**References**

Benson, W. W. et al. 1975. Coevolution of Plants and Herbivores: Passion Flower Butterflies. - Evolution 29: 659–680.

Cormont, A. et al. 2011. Effect of local weather on butterfly flight behaviour, movement, and colonization: significance for dispersal under climate change. - Biodivers Conserv 20: 483–503.

DeVries, P. J. et al. 2010. Vertical distribution, flight behaviour and evolution of wing morphology in Morpho butterflies: Wing evolution in Morpho butterflies. - Journal of Animal Ecology 79: 1077–1085.

Dilts, T. E. et al. 2019. Host Plants and Climate Structure Habitat Associations of the Western Monarch Butterfly. - Front. Ecol. Evol. 7: 188.

Dobkin, D. S. et al. 1987. Rainfall and the interaction of microclimate with larval resources in the population dynamics of checkerspot butterflies (Euphydryas editha) inhabiting serpentine grassland. - Oecologia 71: 161–166.

Hanspach, J. et al. 2014. Host plant availability potentially limits butterfly distributions under cold environmental conditions. - Ecography 37: 301–308.

Karger, D. N. et al. 2017. Climatologies at high resolution for the earth’s land surface areas. - Sci Data 4: 170122.

Karger, D. N. et al. 2019. Climatologies at high resolution for the Earth land surface areas. - Dryad Digital Repository in press.

Kerpel, S. M. et al. 2006. Effect of nitrogen on Passiflora suberosa L. (Passifloraceae) and consequences for larval performance and oviposition in Heliconius erato phyllis (Fabricius) (Lepidoptera: Nymphalidae). - Neotrop. Entomol. 35: 192–200.

Knight, S. M. et al. 2019. Radio-tracking reveals how wind and temperature influence the pace of daytime insect migration. - Biol. Lett. 15: 20190327.

Krämer, B. et al. 2012. Microhabitat selection in a grassland butterfly: a trade-off between microclimate and food availability. - J Insect Conserv 16: 857–865.

Kuussaari, M. et al. 2016. Weather explains high annual variation in butterfly dispersal. - Proc. R. Soc. B 283: 20160413.

O’Donnell, M. S. and Ignizio, D. A. 2012. Bioclimatic predictors for supporting ecological applications in the conterminous United States.: 10.
